# Moisture Controls the Suppression of *Panax notoginseng* Root Rot Disease by Indigenous Bacterial Communities

**DOI:** 10.1128/msystems.00418-22

**Published:** 2022-08-24

**Authors:** Cunwu Guo, Min Yang, Bingbing Jiang, Chen Ye, Lifen Luo, Yixiang Liu, Huichuan Huang, Xinyue Mei, Yifan Zhu, Weiping Deng, Fei Du, Xiahong He, Youyong Zhu, Shusheng Zhu

**Affiliations:** a State Key Laboratory for Conservation and Utilization of Bio-Resources in Yunnan, Yunnan Agricultural University, Kunming, China; b National Engineering Research Center for Applied Technology of Agricultural Biodiversity, College of Plant Protection, Yunnan Agricultural University, Kunming, China; c Key Laboratory for Agro-biodiversity and Pest Control of Ministry of Education, College of Plant Protection, Yunnan Agricultural University, Kunming, China; University of Dundee

**Keywords:** core microbiome, co-occurrence networks, microbial community, *Panax notoginseng*

## Abstract

Harnessing indigenous soil microbial suppression is an emerging strategy for managing soilborne plant diseases. Soil moisture is a vital factor in soil microbiomes, but its role in the regulation of microbial suppression is poorly understood. Here, we investigated the correlation of root rot disease of Panax notoginseng with rhizosphere microbial communities mediated by soil moisture gradients from 55% to 100% field capacity (FC); then, we captured the disease-suppressive and disease-inductive microbiomes and validated their functions by a culture experiment with synthetic microbiotas containing keystone species. We found that proper soil moisture at 75% to 95% FC could maintain a disease-suppressive microbiome to alleviate root rot disease. However, extremely low or high soil moistures (>95% FC or <75% FC) could aggravate root rot disease by depleting the disease-suppressive microbiome while enriching the disease-inductive microbiome. Both the low-soil-moisture-enriched pathogen Monographella cucumerina and the high-soil-moisture-enriched pathogen Ilyonectria destructans could synergize with different disease-inductive microbiomes to aggravate disease. Metagenomic data confirmed that low- and high-moisture conditions suppressed antibiotic biosynthesis genes but enriched pathogenicity-related genes, resulting in a change in the soil state from disease suppressive to inductive. This study highlights the importance of soil moisture when indigenous microbial suppression is harnessed for disease control.

**IMPORTANCE** Soilborne diseases pose a major problem in high-intensity agricultural systems due to the imbalance of microbial communities in soil, resulting in the buildup of soilborne pathogens. Harnessing indigenous soil microbial suppression is an emerging strategy for overcoming soilborne plant diseases. In this study, we showed that soil moisture is a key factor in balancing microbiome effects on root rot disease. Proper soil moisture management represent an effective approach to maintain microbial disease resistance by enriching disease-suppressive microbiomes. Conversely, moisture stresses may enrich for a disease-inductive microbiome and aid accumulation of host-specific soilborne pathogens threatening crop production. This work could provide a new strategy for sustainable control of soilborne diseases by enriching the indigenous disease-suppressive microbiome through soil moisture management.

## INTRODUCTION

Soilborne diseases pose a major problem in high-intensity agricultural systems due to the buildup of pathogens caused by monocultures or relatively short rotations (1). Soilborne diseases are hard to control due to their broad host ranges, mixed infection by multiple pathogens, and the long time required for resistance breeding (2, 3). Although chemical application has been conventionally employed, it is difficult to target the pathogen population in the soil, and this approach can cause environmental contamination (4). The application of biocontrol agents to manage soilborne diseases has become an exciting strategy with which to avoid these issues (5). In the past decade, disease-suppressive soil has been investigated, and culturable antagonistic microbes have been introduced into cropping systems as biocontrol agents for disease management (4, 6, 7). Single-strain biocontrol agents, multiple biocontrol strains, and synthetic bacterial communities have all been applied in the field (4, 5, 8); however, all of these approaches face significant barriers under field conditions due to inadequate colonization (4, 5). In recent years, indigenous antagonistic soil microbiomes with a high ability to adapt to their host and associated environmental conditions have been harnessed to control soilborne diseases (6, 9). The composition and function of the soil microbiome are influenced by many factors, including host species and genotypes, soil characteristics, and environmental conditions (9–12). Among edaphic factors, soil moisture is the most important and easily managed and the most neglected factor. Low or high soil moisture, frequently resulting from climate extremes, affects plant health during plant production (13, 14).

Many previous studies have reported that soil moisture stress, as a major factor, results in the deterioration of plant physiology and then susceptibility to disease (15, 16) or favors the growth and reproduction of host-specific pathogens (12, 17–19). Although many recent studies have documented that drought or waterlogging causes major changes to the soil microbial community (19–23), their effect on soilborne disease suppression has not been reported. Thus, it is important to investigate the association of the soil microbiome with soilborne-disease dynamics and its regulation by soil moisture from the perspective of microbial ecology.

Panax notoginseng is a typical shade-demanding plant that is sensitive to soil moisture changes and faces severe root rot disease when it is infected by soilborne pathogens (24, 25). Many studies have shown that the occurrence of root rot disease in *P. notoginseng* is greatly influenced by the soil microbial community, which is modified by host development and soil physicochemical properties (26–28). Moreover, it is usually observed that a high disease incidence occurs when the soil moisture is too high or too low in the field. We presumed that the occurrence of root rot disease could be affected by the soil microbiome, which is influenced by soil moisture. However, it is still unknown how the microbial community and its ability to suppress soilborne pathogens are regulated by soil moisture. Therefore, we chose *P. notoginseng* as a model plant to (i) analyze the correlation of root rot disease with the rhizosphere microbiome mediated by soil moisture from low to high, (ii) capture the keystone species associated with disease suppression using a network based on correlation analysis and validate the role of the keystone species by analyzing *in planta* and *in vitro* interaction with a culturable synthetic community, and (iii) determine the functional profiles of soil microbial suppression affected by soil moisture.

## RESULTS

### Soil moisture stress aggravates root rot disease.

**FIG 1:**
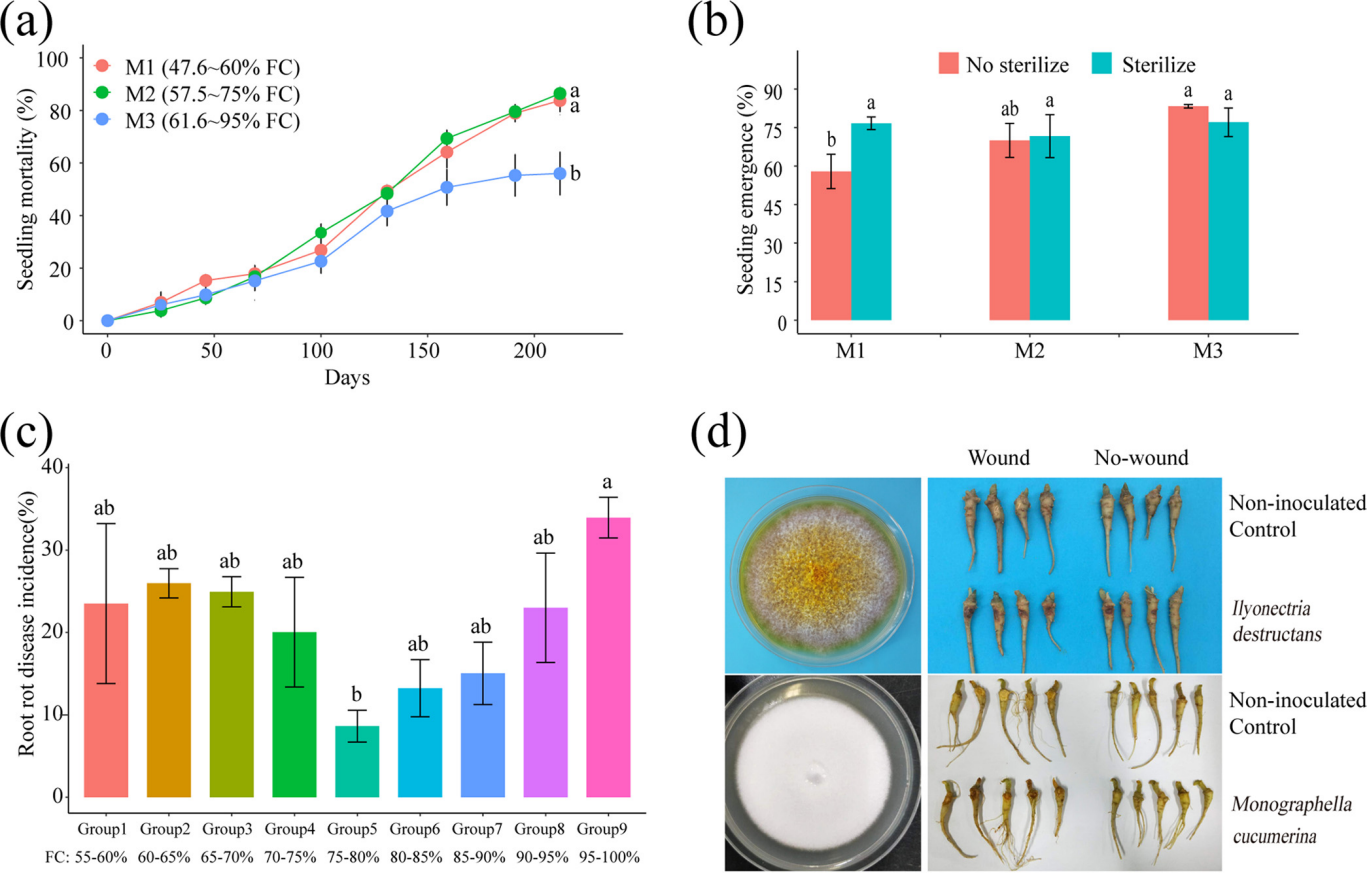
Effect of soil moisture on root rot disease of *Panax notoginseng* in field and pot experiments. (a) Seedling mortality at soil moistures M1 (47.6 to 60% FC), M2 (57.5 to 75% FC), and M3 (61.6 to 95%) observed for 7 months. (b) Seeding emergence rate in continuous cropping soil samples with three moisture levels (M1, M2, and M3) with or without sterilization (90°C for 15 min). (c) Root rot disease incidence at the nine levels of soil moistures from low to high in the pot experiment. Group 1, 55 to 60% FC; group 2, 60 to 65% FC; group 3, 65 to 70% FC; group 4, 70 to 75% FC; group 5, 75 to 80% FC; group 6, 80 to 85% FC; group 7, 85 to 90% FC; group 8, 90 to 95% FC; group 9, 95 to 100% FC. (d) Pathogenic fungi *Ilyonectria destructans* and *Monographella cucumerina* isolated from root rot tissues and identified using Koch’s postulates. Data are means and standard errors (SE: error bars). Different letters indicate significant differences (*P < *0.05; ANOVA, Tukey’s HSD test) among the treatments (*n* = 4 for each treatment in the field, *n* = 3 for each treatment in the pot experiment).

In the field experiment, plant mortality significantly increased with low soil moisture (M1, 47.6 to 60% field capacity [FC], and M2, 57.5 to 75% FC) compared with that with high soil moisture (M3: 61.6 to 95% FC) after 7 months of continuous soil moisture control (Fig. 1a; also, see Fig. S1a in the supplemental material). When seedlings were replanted in continuous cropping soil from the three treatments, the seedling germination rate significantly decreased in the low-soil-moisture treatment (M1) compared with that in the high-soil-moisture treatment (M3) (Fig. 1b). When the continuous cropping soil was steamed at 90°C for 15 min, the difference in seedling germination rates among the three treatments disappeared (Fig. 1b). These data indicated that soil moisture could modify the soil microbiome to affect plant health.

10.1128/msystems.00418-22.1 FIG S1 Soil moisture treatments in pot and field experiments. (a) Three levels of soil moisture were continuously controlled for 7 months in the field experiment. The soil moisture was manually adjusted to the appropriate level every 48 h and monitored with time domain reflectometry (TDR 100 soil moisture meter). (b) Nine levels of soil moisture, ranging from 55% FC to 100% FC, were used in the pot experiment. The soil moisture was maintained by manual irrigation every 48 h and monitored using the accurate weight method. Download FIG S1, TIF file, 0.7 MB. Copyright © 2022 Guo et al. 2022 Guo et al. https://creativecommons.org/licenses/by/4.0/ This content is distributed under the terms of the Creative Commons Attribution 4.0 International license.

In soil moisture-controlled pot experiments (Fig. S1b), low- and high-soil-moisture treatments all aggravated the occurrence of root rot disease (Fig. 1c). In particular, the incidence of root rot disease significantly increased in both the low-soil-moisture group (group 2, 60% to 65% of FC) and high-soil-moisture group (group 9, 95 to 100% of FC) compared with that of the middle-soil-moisture group (group 5, 75 to 80% of FC) (Fig. 1c). Ilyonectria destructans and Monographella cucumerina were frequently isolated from rotten roots and identified as destructive pathogens by Koch’s postulates (Fig. 1d). This pot experiment further confirmed that proper soil moisture was suitable for plant health, but soil moisture stress, including low and high soil moisture, could aggravate root rot disease.

### Soil moisture modified the microbial community to affect root rot disease.

**FIG 2:**
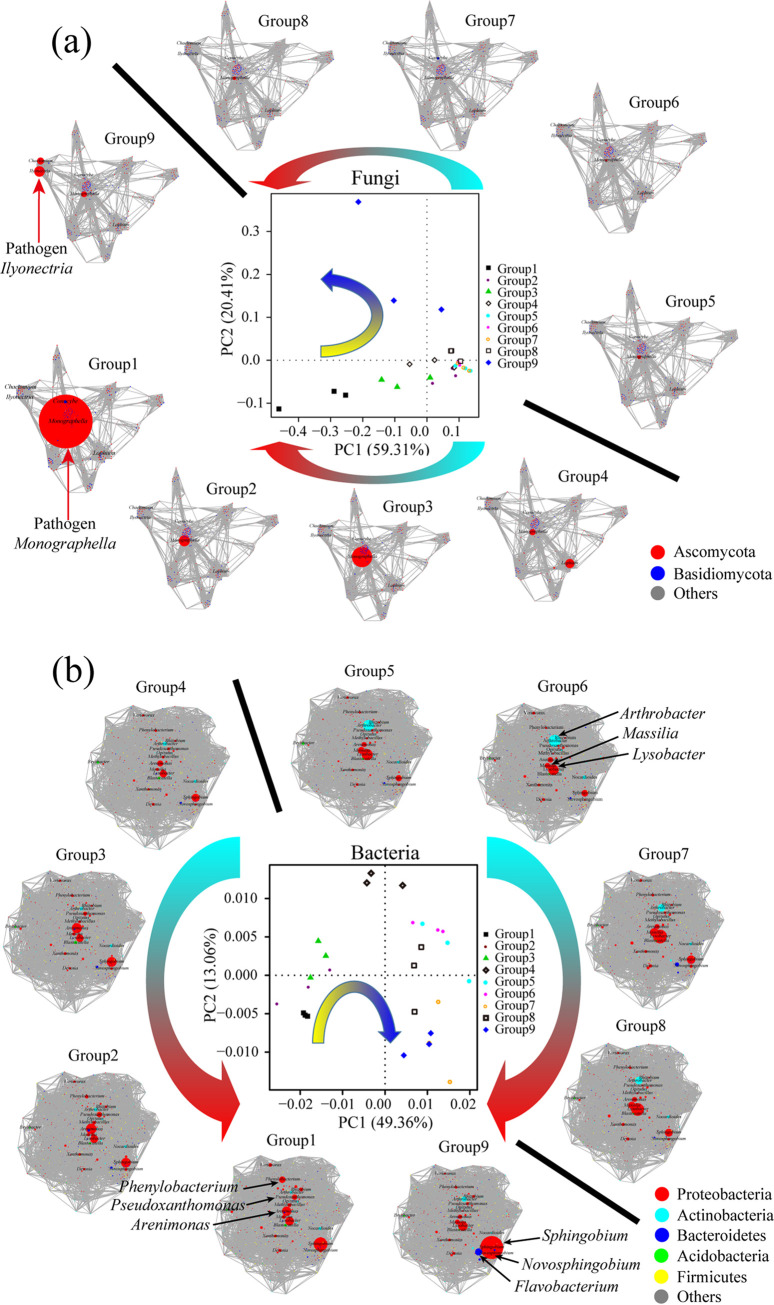
Effects of soil moisture on rhizosphere microbial community and coabundance network. (a) Fungal community and coabundance networks affected by soil moisture. The PCoA plots show the nine soil moisture treatments defined by the Bray-Curtis analysis of the fungal microbiome community (middle). In the coabundance networks, each node represents an individual fungal genus, the node size is proportional to the mean relative abundance in each treatment, and the connections between the nodes represent Pearson correlations (*r* > 0.6 or *r* < −0.6 or *P < *0.05) between genera. The red and blue colors in the plots represent individual genera from *Ascomycota* and *Basidiomycota*, respectively. (b) Bacterial community and coabundance networks affected by soil moisture. The PCoA plots show nine soil moisture treatments defined by a weighted UniFrac analysis of the bacterial microbiome community (middle). The individual genera from *Proteobacteria* (red), *Actinobacteria* (cyan), *Bacteroidetes* (blue), *Acidobacteria* (green) and *Firmicutes* (yellow) are shown in the plots. The arrows in the PCoA plots indicate the direction of change in the fungal and bacterial communities from low soil moisture (yellow) to high soil moisture (blue). Arrows outside the PCoA plots indicate the transition of soilborne disease incidence from low (cyan) to high (red).

Principal-coordinate analysis (PCoA) showed that fungal and bacterial communities clearly separated the groups with the change in soil moisture from low to high (Fig. 2a and b). The incidences of root rot disease in the nine soil moisture treatments were significantly negatively correlated with the first axis of the PCoA for fungi (Spearman correlation; *r =* −0.783, *P = *0.013) and bacteria (Spearman correlation; *r =* −0.667, *P = *0.04999). In addition, the incidences of disease in the nine soil moisture treatment groups were significantly positively correlated with the Simpson (Pearson correlation; *r = *0.673, *P = *0.047) and Shannon (Pearson correlation; *r = *0.696, *P = *0.037) indices of the fungi (Fig. S2a and b) but negatively correlated with the Simpson (Pearson correlation; *r =* −0.639, *P = *0.064) and Shannon (Pearson correlation; *r =* −0.556, *P = *0.120) indices of the bacteria (Fig. S2c and d).

10.1128/msystems.00418-22.2 FIG S2 Alpha diversity indices and relative abundances (top 10) at the phylum level of the fungi and bacteria in the rhizosphere soil affected by different levels of soil moisture. (a and b) Shannon and Simpson indices of fungi from the rhizosphere soil of *P. notoginseng* with different levels of soil moisture in the pot experiment. (c and d) Shannon and Simpson indices of bacteria from the rhizosphere soil of *P. notoginseng* with different levels of soil moisture in the pot experiments. (e and f) Hierarchical clustering analysis of the relative abundances (top 10) of the fungi (e) and bacteria (f) at the phylum level. The horizontal bars within the boxes represent the medians. The tops and bottoms of the boxes represent the 75th and 25th percentiles, respectively. The upper and lower whiskers extend to data no more than 1.5× the interquartile range from the upper edge and lower edge of the box, respectively. The *r* and *P* values in the plot represent the relationship and significance between the alpha diversity index and the root rot disease incidence, respectively. The different letters indicate significant differences (*P < *0.05; ANOVA, Tukey’s HSD test) among the treatments (*n* = 3 for each treatment). The asterisk indicates a statistically significant difference (*P < *0.05; ANOVA, Tukey’s HSD test) among the nine soil water treatments (*n* = 3 for each treatment). Download FIG S2, TIF file, 1.0 MB. Copyright © 2022 Guo et al. 2022 Guo et al. https://creativecommons.org/licenses/by/4.0/ This content is distributed under the terms of the Creative Commons Attribution 4.0 International license.

Coabundance network analysis of the fungi revealed the enrichment of pathogenic *Monographella* under low soil moisture (groups 1 to 4, 55% to 75% of FC) and pathogenic *Ilyonectria* under high soil moisture (group 9, 95% to 100% of FC) (Fig. 2a). Importantly, the incidence of root rot disease in different treatments was positively correlated with the relative abundance of *Ilyonectria* (Pearson correlation, *r = *0.680, *P = *0.044) and *Monographella* (Pearson correlation, *r = *0.296, *P = *0.440) (Table S1A). The coabundance network of bacteria revealed that the top 10 genera with the highest relative abundance, including *Sphingobium*, *Novosphingobium*, *Flavobacterium*, and *Sphingopyxis*, which were significantly enriched in the high-soil-moisture treatment (group 9, 95% to 100% of FC) (*P < *0.05) (Fig. 2b). Among them, *Sphingobium*, *Novosphingobium*, and *Sphingopyxis* were significantly positively correlated with the incidence of root rot disease (Table S1B). Some genera, including *Arenimonas*, *Phenylobacterium*, and *Pseudoxanthomonas*, were significantly enriched in the low-soil-moisture treatments (groups 1 and 2, 55% to 65% of FC) (*P < *0.05) (Fig. 2b). However, some genera, including *Lysobacter*, *Arthrobacter*, and *Massilia*, were significantly enriched in the treatments with soil moisture ranging from 75% to 80% of FC (*P < *0.05) (Fig. 2b). Among them, *Arthrobacter* was significantly negatively correlated with the incidence of root rot disease (Table S1B).

10.1128/msystems.00418-22.6 TABLE S1 (A) Relationship between genus-level relative abundance from Illumina sequencing of the ITS and root rot disease incidence. (B) Relationship between genus-level relative abundance from Illumina sequencing of the 16S rRNA gene and soilborne disease incidence. Download Table S1, XLSX file, 0.05 MB. Copyright © 2022 Guo et al. 2022 Guo et al. https://creativecommons.org/licenses/by/4.0/ This content is distributed under the terms of the Creative Commons Attribution 4.0 International license.

### Soil moisture-modified keystone species associated with root rot disease.

**FIG 3:**
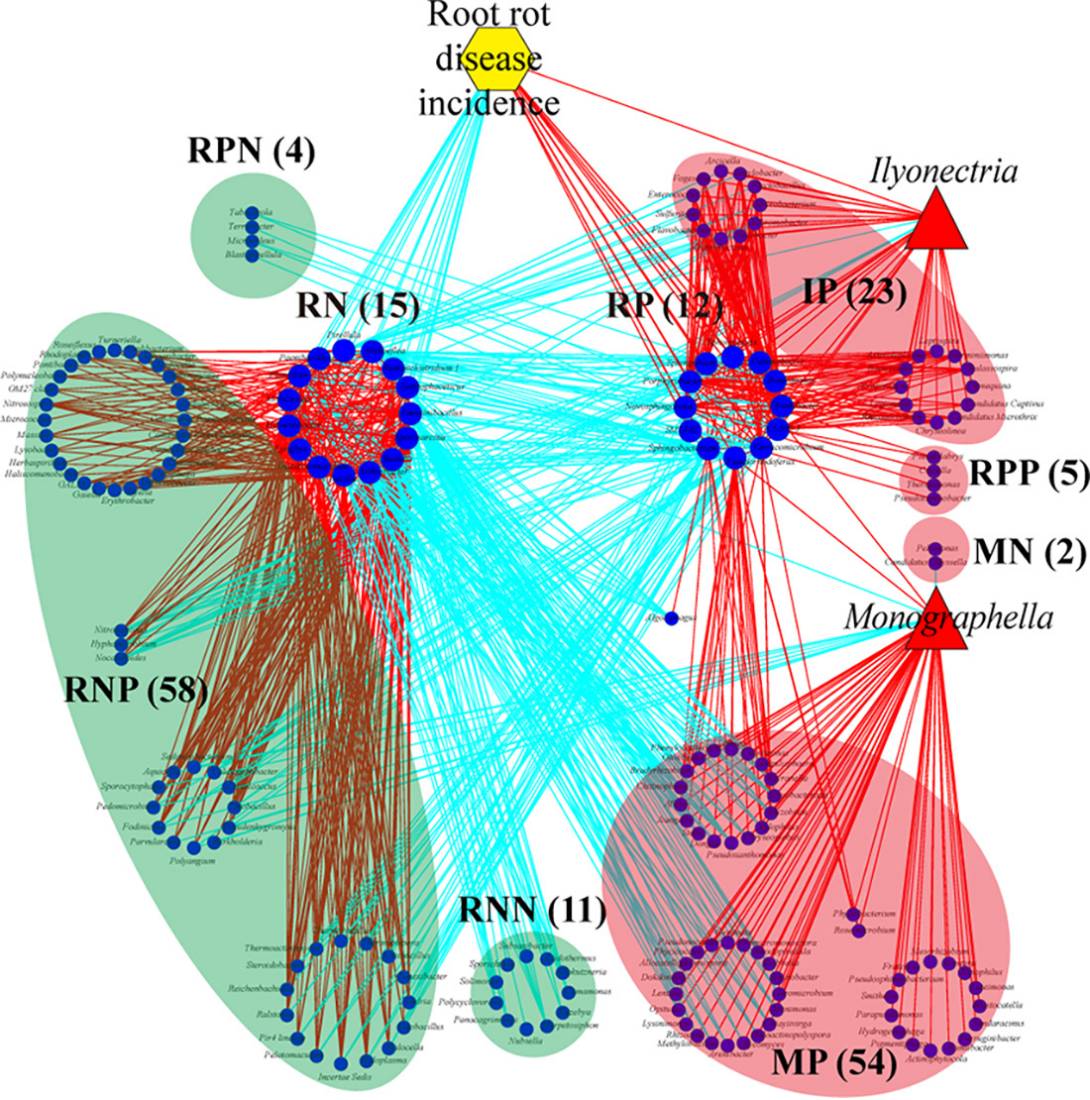
Networks of the keystone bacterial species associated with soilborne disease and two fungal pathogens (*Ilyonectria* and *Monographella*). The connections between the nodes represent significant Pearson correlations (*P < *0.05). The cyan and red lines represent negative and positive connections between the nodes, respectively. The blue circles and red triangles represent the genera of bacteria and fungi, respectively, and the yellow hexagon represents the soilborne-disease incidence. All genera that were significantly negatively correlated with soilborne disease were defined as belonging to the RN community. All genera that were significantly positively or negatively correlated with the RN community were defined as belonging to the RNP and RNN communities, respectively. All genera that were significantly positively correlated with soilborne disease were defined as belonging to the RP community. All genera that were significantly positively or negatively correlated with the RP community were defined as belonging to the RPP and RPN communities, respectively. All genera that were significantly positively correlated with *Ilyonectria* and *Monographella* were defined as belonging to the IP and MP communities, respectively. Genera that showed only a significant negative correlation with the *Monographella* were defined as members of the MN community. The numbers in the brackets show the numbers of all the genera in each community.

To further identify the keystone species associated with root rot disease, the correlations of all 266 bacterial genera that were significantly modified by soil moisture (Table S2E) with root rot disease and the pathogenic fungi *Ilyonectria* and *Monographella* were further analyzed. A total of 15 keystone genera were significantly negatively correlated with root rot disease incidence (defined as the RN microbiota), and 12 keystone genera were significantly positively correlated with root rot disease (defined as the RP microbiota) (Fig. 3). The remaining genera, which were correlated with RN, RP, *Ilyonectria*, or *Monographella*, were separated into seven groups (Fig. 3). All genera that were significantly positively or negatively correlated with the RN community were defined as belonging to the RNP and RNN communities, respectively. All genera that were significantly positively or negatively correlated with the RP community were defined as belonging to the RPP and RPN communities, respectively. All genera that were significantly positively correlated with Ilyonectria and Monographella were defined as belonging to the IP and MP communities, respectively. Genera that showed only a significant negative correlation with the Monographella were defined as members of the MN community. Among them, the RNP microbiota (58 genera) showed a significant positive correlation with the RN microbiota. The IP microbiota (23 genera) and MP microbiota (54 genera) showed a significant positive correlation with pathogenic *Ilyonectria* and *Monographella*, respectively (Fig. 3). Therefore, we inferred that two opposite groups, including the disease-suppressive microbiome (RN, RNP, MN, and RPN) and the disease-inductive microbiome (RP, IP, MP, RPP, and RNN), existed in rhizosphere soil based on their correlation with root rot disease (Fig. 3).

10.1128/msystems.00418-22.7 TABLE S2 (A) Number of reads and alpha-diversity indices from Illumina sequencing of the 16S rRNA gene from rhizosphere soil. (B) Number of reads and alpha-diversity indices from Illumina sequencing of the ITS gene from rhizosphere soil. (C) Difference analysis of the relative abundance of bacterial phyla among different soil moisture treatments. (D) Difference analysis of the relative abundance of fungal phyla among different soil moisture treatments. (E) Bacterial genera significantly mediated by soil moisture based on Illumina sequencing of the 16S rRNA gene. Download Table S2, XLSX file, 0.04 MB. Copyright © 2022 Guo et al. 2022 Guo et al. https://creativecommons.org/licenses/by/4.0/ This content is distributed under the terms of the Creative Commons Attribution 4.0 International license.

**FIG 4:**
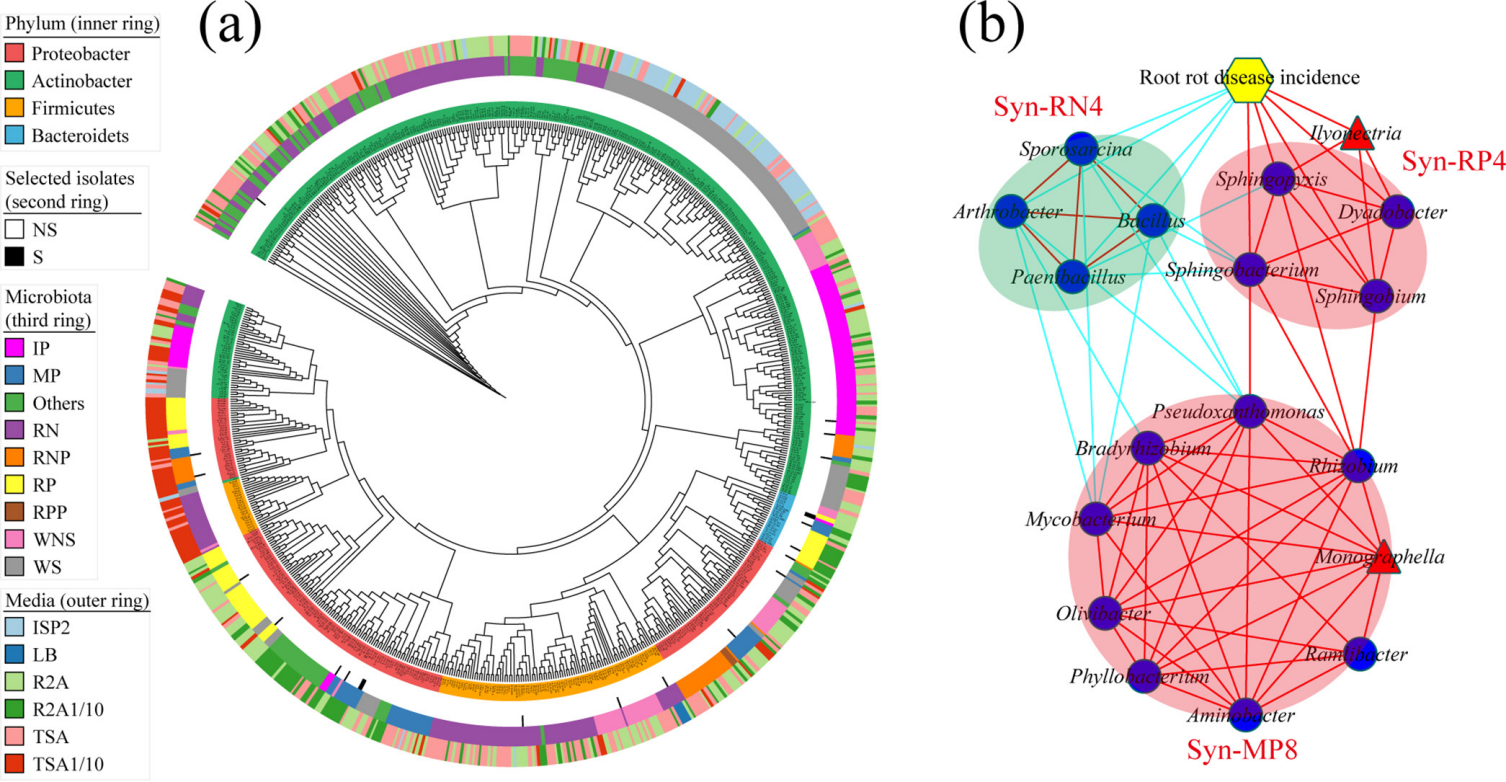
Phylogenetic tree of 734 bacterial isolates from the rhizosphere soil and three synthetic microbiotas associated with soilborne disease. (a) Cladogram showing the phylogenetic tree of the 734 bacterial isolates from the rhizosphere soil and their origins. The leaf labels indicate the representative sequence IDs. The rings, from the inner to the outer circles, represent the isolates belonging to the phylum, the isolates selected to form the synthetic microbiotas (NS, not selected; S, selected), the isolates belonging to the community, and the medium on which the isolates were originally obtained. Detailed information for all isolates is provided in Table S3. (b) Construction of three synthetic microbiotas based on the culturable bacterial isolates. Syn-RN4 consists of four isolates from the genera *Arthrobacter*, *Bacillus*, *Paenibacillus*, and *Sporosarcina*; Syn-RP4 consists of four isolates from the genera *Dyadobacter*, *Sphingobacterium*, *Sphingobium*, and *Sphingopyxis*; Syn-MP8 consists of eight isolates from the genera *Rhizobium*, *Bradyrhizobium*, *Aminobacter*, *Phyllobacterium*, *Olivibacter*, *Pseudoxanthomonas*, Mycobacterium, and *Ramlibacter*. The connections between the nodes represent significant Pearson correlations between the genera (*P < *0.05). The cyan and red lines represent negative connections and positive connections between two nodes, respectively. The blue circles and red triangles represent the genera of bacteria and fungi, respectively, and the yellow hexagon represents the soilborne-disease incidence. WS represent others isolating rhizosphere bacterial genera were significantly modified by soil moisture from the Illumina sequencing, WNS represent the isolating rhizosphere bacterial genera were no significantly modified by soil moisture from the Illumina sequencing, Others represent the isolating rhizosphere bacterial genera have not detected from the Illumina sequencing.

To confirm the role of keystone species in root rot disease, a total of 734 bacterial isolates belonging to 66 genera originating from four phyla were isolated from the rhizosphere soil (Fig. 4a; Table S3B). Among them, a total of 28 genera belonging to the RN, RP, MP, RNP, IP, and RPP microbiotas were isolated (Fig. 4a; Table S3B). All 201 isolates in the RN microbiota belonged to *Actinobacteria* and *Firmicutes* (Fig. 4a; Table S3A), and most of them showed antagonistic activity against *I. destructans* and *M. cucumerina* (Table S3A). However, most of the isolates in the RP, IP, MP, and RPP microbiotas belonged to *Proteobacteria* and *Bacteroidetes* (Fig. 4a; Table S3A). Most of these isolates did not show antagonistic activity against *I. destructans* and *M. cucumerina* (Table S3C to E).

10.1128/msystems.00418-22.8 TABLE S3 (A) Information on 734 bacterial isolates aligned against NCBI and isolated from *Panax notoginseng* rhizosphere soil by culture experiment. (B) Classification of 734 bacterial strains. (C) Sorting of 734 isolates against *I. destructans* based on antagonistic activities. (D) Sorting of 734 isolates against *M. cucumerina* based on antagonistic activities. (E) Antagonistic activities of 414 bacterial strains isolated against *I. destructans* and *M. cucumerina*. Download Table S3, XLSX file, 0.07 MB. Copyright © 2022 Guo et al. 2022 Guo et al. https://creativecommons.org/licenses/by/4.0/ This content is distributed under the terms of the Creative Commons Attribution 4.0 International license.

**FIG 5:**
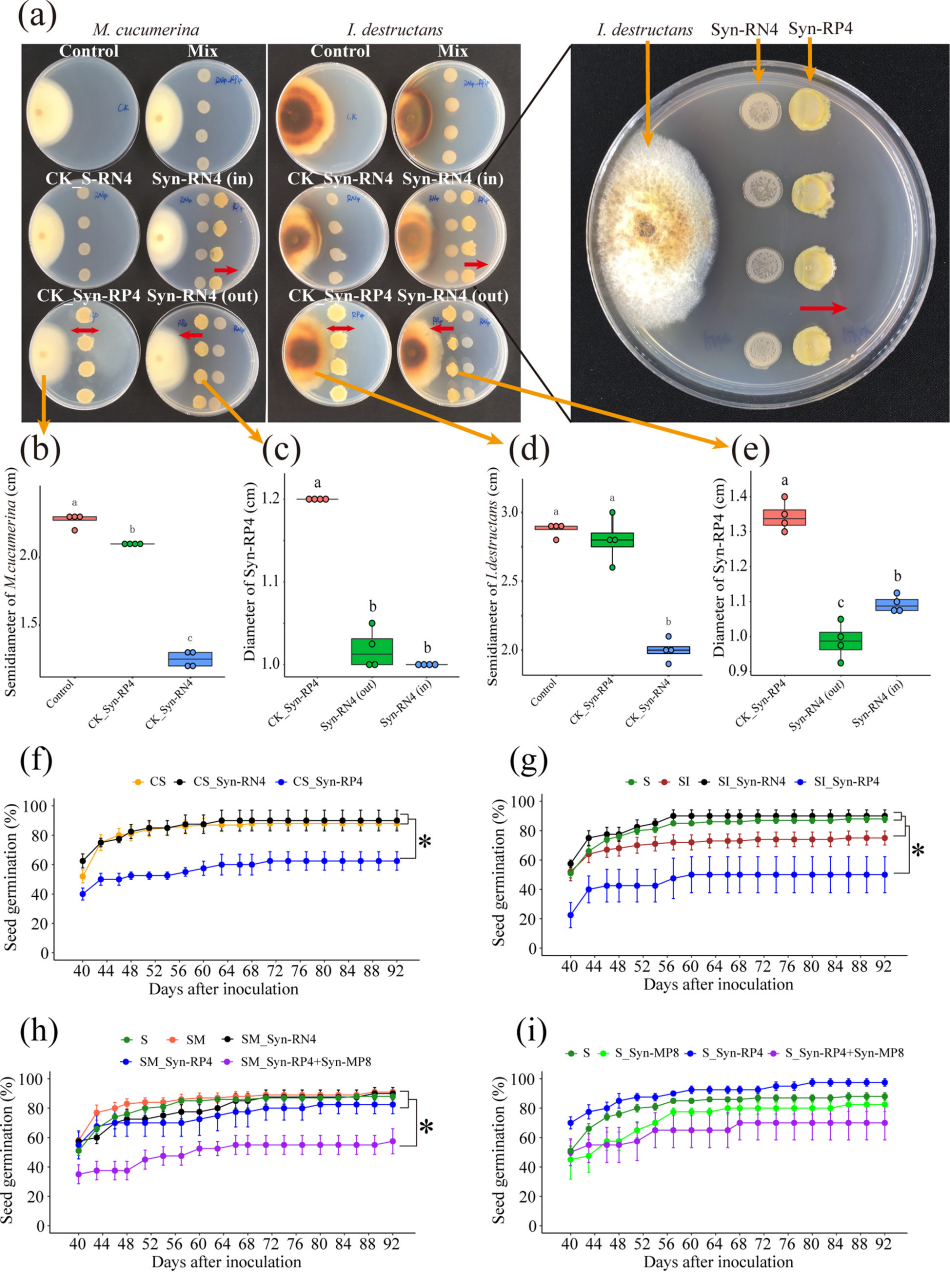
Antagonistic activity of the synthetic Syn-RN4 and Syn-RP4 bacterial microbiotas against soilborne pathogens and their effects on soilborne-disease occurrence. (a) Antagonistic activity between Syn-RN4 and Syn-RP4 or against the two pathogenic fungi (*I. destructans* and *M. cucumerina*) in petri dishes. The growth direction of Syn-RP4 was far from that of Syn-RN4 (red arrows). (b and c) Antagonistic activity of Syn-RN4 and Syn-RP4 against *M. cucumerina*. (d and e) Antagonistic activity of Syn-RN4 and Syn-RP4 against *I. destructans* or each other. The horizontal bars within the boxes represent the medians. The tops and bottoms of boxes represent the 75th and 25th percentiles, respectively. The upper and lower whiskers extend to data no more than 1.5× the interquartile range from the upper edge and lower edge of the box, respectively. The different letters indicate significant differences (*P < *0.05; ANOVA, Tukey’s HSD test) among the treatments (*n* = 4 for each treatment). (f) Seed germination rate when Syn-RP4 or Syn-RN4 was added to the continuous cropping soil (CS soil); CS_Syn-RN4 represents Syn-RN4 communities added to CS soil; CS_Syn-RP4 represents Syn-RP4 communities added to CS soil. (g) Seed germination rate when Syn-RP4 or Syn-RN4 was added to the SI soil. S represents sterilized soil; SI represents sterilized soil inoculated with *I. destructans*; SI_Syn-RN4 represents Syn-RN4 communities added to SI soil; SI_Syn-RP4 represents Syn-RP4 communities added to SI soil. (h) Seed germination rate when Syn-RP4, Syn-RN4, or a mixture of Syn-RP4 and Syn-MP8 was added to SM soil (sterilized soil inoculated with *M. cucumerina*; represented by SM_Syn-RP4, SM_Syn-RN4, and SM_Syn-RP4+Syn-MP8, respectively). (i) Seed germination rate when Syn-RP4, Syn-RN4, or a mixture of Syn-RP4 and Syn-MP8 was added to the S soil (represented by S_Syn-RP4, S_Syn-RN4, and S_Syn-RP4+Syn-MP8, respectively). Dots represent the medians. Error bars indicate standard errors. The asterisk indicates a statistically significant difference (*P* < 0.05; ANOVA, Tukey’s HSD test) among the treatments (*n* = 4 for each treatment, except *n* = 10 for the control).

A total of 16 isolates from 16 genera were further selected as keystone species to form three synthetic microbiotas (Syn-RN4, Syn-RP4, and Syn-MP8) (Fig. 4b). Syn-RN4 showed higher antagonistic activity against the colony growth of *I. destructans* than its individual isolates (Fig. S3a). Syn-RP4 showed a higher ability to inhibit seed germination than its individual isolates (Fig. S3e to g). Therefore, the antagonistic activity of Syn-RN4 and Syn-RP4 against soilborne pathogens and their effect on root rot disease were further tested. Syn-RN4 showed antagonistic activity against the growth of Syn-RP4 as well as that of the pathogens (*I. destructans* and *M. cucumerina*) (Fig. 5a, b and d). In particular, Syn-RN4 affected the growth direction of Syn-RP4 from a great distance (Fig. 5a) and decreased the growth diameter of Syn-RP4 (Fig. 5c and e). Syn-RP4 did not suppress the growth of *M. cucumerina* and *I. destructans* (Fig. 5d).

10.1128/msystems.00418-22.3 FIG S3 (a) Antagonistic activity of two synthetic microbiota (Syn-RN4 and Syn-RP4) and their individual isolates against the growth of two soilborne pathogens (*Ilyonectria* and *Monographella*). (b to d) Differences in the seed germination rate among Syn-RN4 and its individual isolates (A, B, P, and S) in the CS, SI, and SM soils. (e to g) Differences in the seed germination rate among Syn-RP4 and its individual isolates (D, Sba, Sbi, and Spy) in the CS, SI, and SM soils. CS, continuous cropping soil; SI, sterilized continuous cropping soil inoculated with *I. destructans*; SM, sterilized continuous cropping soil inoculated with *M. cucumerina.* A, *Arthrobacter*; B, *Bacillus*; P, *Paenibacillus*; S, *Sporosarcina*; D, *Dyadobacter*; Sba, *Sphingobacterium*; Sbi, *Sphingobium*; Spy, *Sphingopyxis*; Syn-RN4, mixture of A, B, P, and S isolates; Syn-RP4, mixture of D, Sba, Sbi, and Spy isolates. The horizontal bars within the boxes represent the medians. The tops and bottoms of the boxes represent the 75th and 25th percentiles, respectively. The upper and lower whiskers extend to data no more than 1.5× the interquartile range from the upper edge and lower edge of the box, respectively (a, b, c, and d). The different letters indicate significant differences among the treatments (*P < *0.05; ANOVA, Tukey’s HSD test). Download FIG S3, TIF file, 3.0 MB. Copyright © 2022 Guo et al. 2022 Guo et al. https://creativecommons.org/licenses/by/4.0/ This content is distributed under the terms of the Creative Commons Attribution 4.0 International license.

When Syn-RP4 was added to the continuous cropping soil, the seed germination rate was significantly decreased compared with that of the control continuous cropping soil (CS soil) (Fig. 5f). The seed germination rate in the sterilized soil inoculated with *I. destructans* (SI soil) was lower than that in the sterilized soil (S soil) (Fig. 5g). When Syn-RP4 was inoculated into SI soil, the seed germination rate further decreased (Fig. 5g). Syn-RN4 addition increased the seed germination rate compared with that in the SI soil (Fig. 5g). However, there was no significant decrease in seed germination when Syn-RP4 was added to the soil inoculated with *M. cucumerina* (SM soil) (Fig. 5h). Interestingly, the seed germination rate was significantly decreased when a mixture of Syn-RP4 and Syn-MP8 was added to the SM soil (Fig. 5h). In the sterilized soil, however, Syn-RP4, Syn-MP8 and Syn-RP4+Syn-MP8 did not show any significant difference in seed germination compared with that in the S soil control (Fig. 5i). These data confirmed that Syn-RP4 could synergize with *I. destructans* and that Syn-MP8 could synergize with *M. cucumerina* to aggravate disease. However, Syn-RN4 suppressed Syn-RP4 and Syn-MP8 as well as soilborne pathogens to alleviate disease.

### Imbalance of disease-suppressive and disease-inductive microbiomes caused by soil moisture stress aggravates root rot disease.

**FIG 6:**
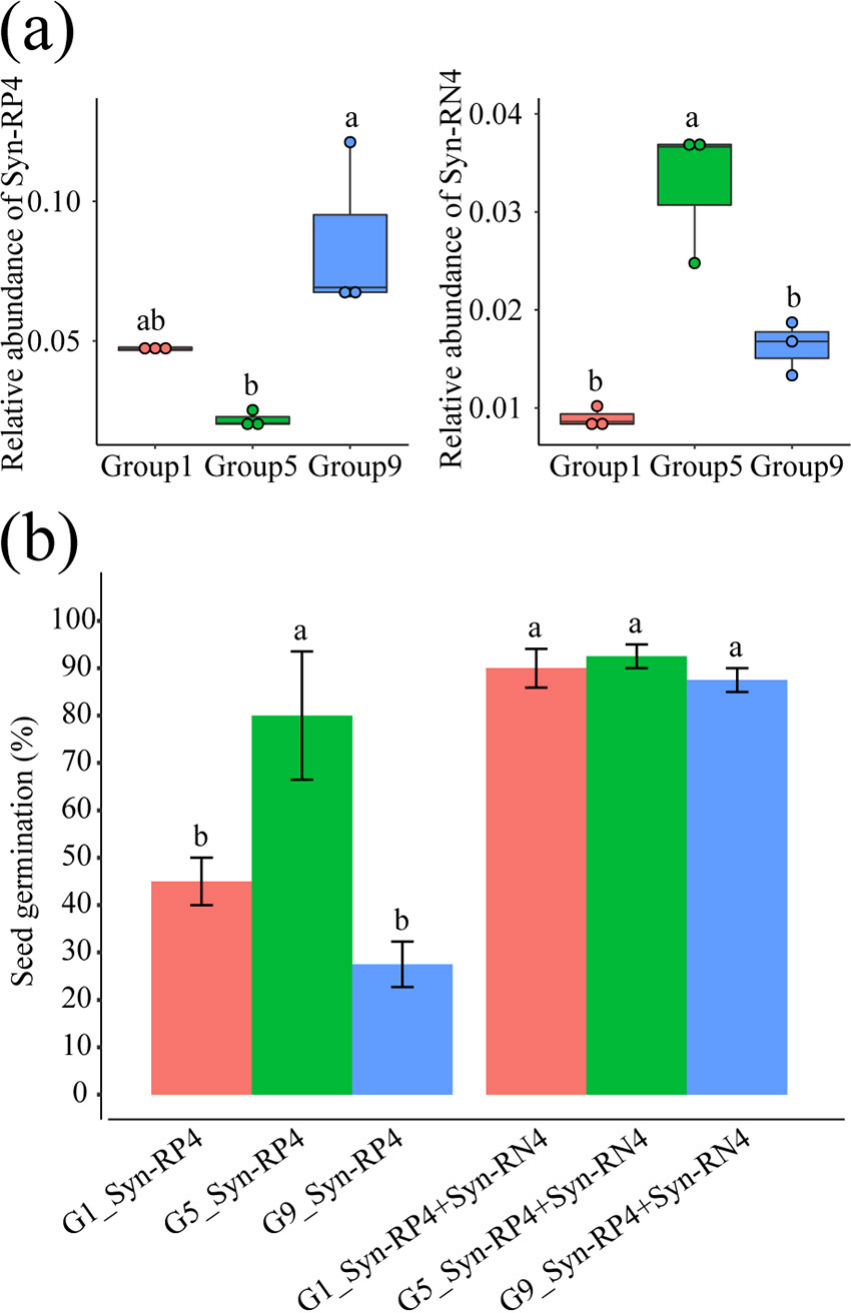
Effects of the change of Syn-RN4 and Syn-RP4 communities in soil on the seedling emergence of *Panax notoginseng*. (a) Total relative abundance of Syn-RP4 and Syn-RN4 in group 1, group 5, and group 9. (b) Seedling emergence rate when the Syn-RP4 community was inoculated individually or together with Syn-RN4 into sterilized soil according to their relative abundances in group 1, group 5, and group 9 treatments. G1_Syn-RP4, G5_Syn-RP4, and G9_Syn-RP4, respectively, represent Syn-RP4 inoculated into sterilized soil according to their relative abundances in group 1, group 5, and group 9 treatments; G1_Syn-RP4+Syn-RN4, G5_Syn-RP4+Syn-RN4, and G9_Syn-RP4+Syn-RN4, respectively, represent Syn-RN4 together with Syn-RP4 inoculated into sterilized soil according to their relative abundances in group 1, group 5, and group 9 treatments. The error bars indicate the standard errors. Asterisks indicate statistically significant differences (*P < *0.05; ANOVA, Tukey’s HSD test) among the treatments (*n* = 4 for each treatment).

We further added Syn-RP4 and Syn-RN4+Syn-RP4 into continuous cropping soil according to their relative abundances in low soil moisture (group 1, 55 to 60% of FC), proper soil moisture (group 5, 75 to 80% of FC), and high soil moisture (group 9, 95 to 100% of FC) (based on 16S rRNA amplicon sequencing) (Fig. 6a). With the increase in the relative abundance of Syn-RP4, the seed germination rate was significantly decreased (Fig. 6b). When Syn-RN4 was added to the soil together with Syn-RP4 (Syn-RP4+Syn-RN4), the seed germination rate was significantly increased (Fig. 6b). The seed germination rate was the highest in the G5_Syn-RP4+Syn-RN4 treatment compared with that in the G1_Syn-RP4+Syn-RN4 and G9_Syn-RP4+Syn-RN4 treatments, although there was no significant difference (Fig. 6b). This further confirmed that low and high soil moisture stress could enrich the indigenous disease-inductive microbiome to aggravate root rot disease. However, proper soil moisture could maintain an indigenous disease-suppressive microbiome to alleviate root rot disease.

### Soil moisture stress suppresses antibiotic biosynthesis genes but enriches pathogenicity-related genes.

**FIG 7:**
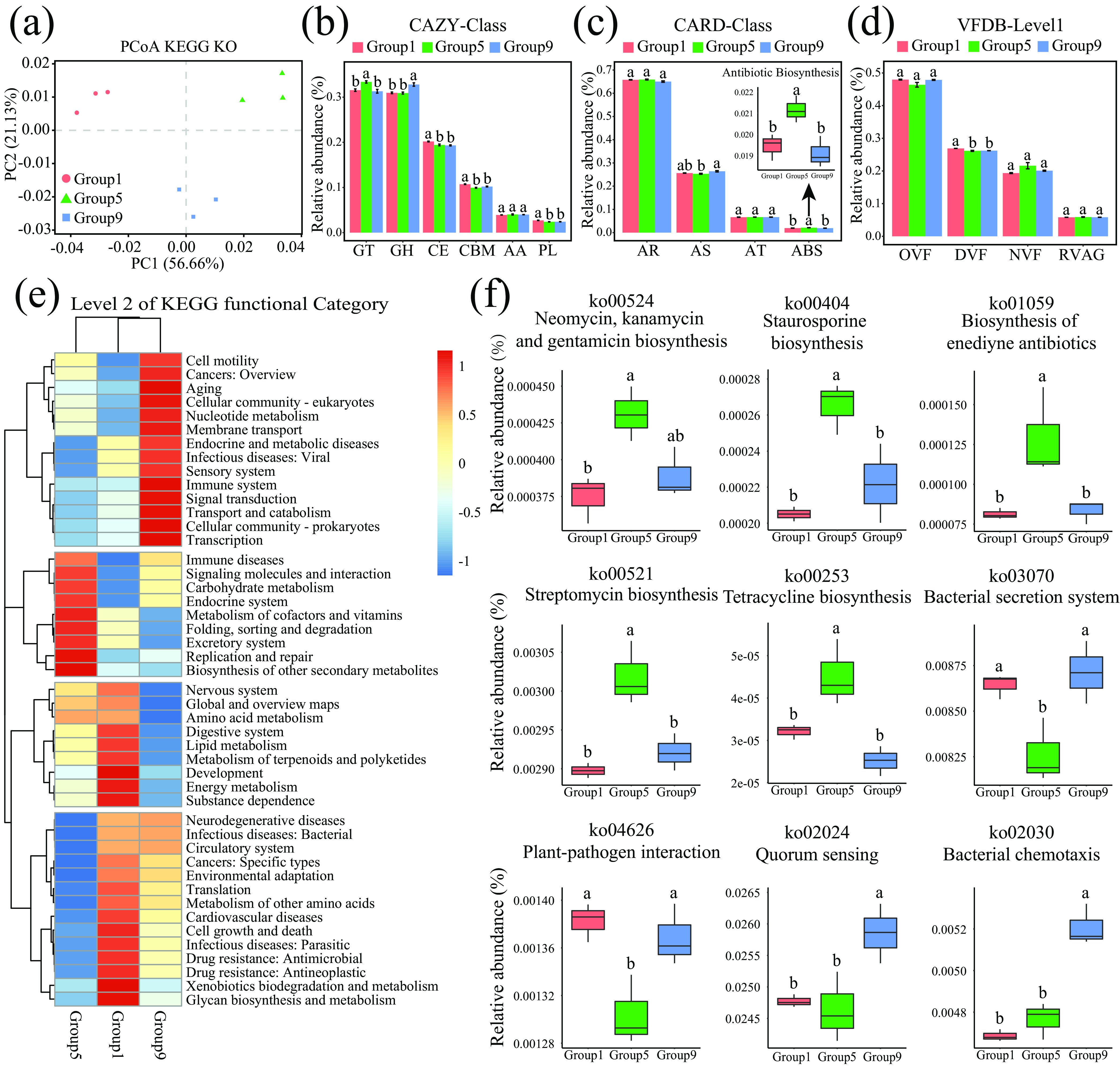
Effect of soil moisture on microbial function based on metagenomic shotgun sequencing. (a) PCoA based on the KEGG, showing clear separation among the three treatments (group 1, group 5, and group 9). (b to d) Relative abundances of the genes annotated with the CAZy, CRDB, and VFDB databases among the three treatments. (e) Heat map of the level 2 KEGG functional categories. The columns represent the three treatments. (f) Some pathways involved in antibiotic biosynthesis and plant-pathogen interactions showed significant differences among the three treatments. The horizontal bars within the boxes represent the medians. The tops and bottoms of the boxes represent the 75th and 25th percentiles, respectively. The upper and lower whiskers extend to data no more than 1.5× the interquartile range from the upper edge and lower edge of the box, respectively. The different letters indicate significant differences (*P < *0.05; ANOVA, Tukey’s HSD test) among the treatments (*n* = 3 for each treatment).

The total DNA of the rhizosphere soil microbes from three treatments, a low-soil-moisture treatment (group 1, 55 to 60% of FC), a proper-soil-moisture treatment (group 5, 75 to 80% of FC), and a high-soil-moisture treatment (group 9: 95 to 100% of FC), were analyzed using metagenomic shotgun sequencing. Based on KEGG database annotation, PCoA showed clear separation among the three treatments (Fig. 7a). Further PCoA based on CAZy, CARD, ARDB, VFDB, NR, QS, PHI, and P450 database annotations were also clearly separated among the three treatments (Fig. S4). Genes involved in glycosyl transferases (CAZy database) (Fig. 7b) and antibiotic biosynthesis (CARD database) (Fig. 7c; Table S4D) were significantly enriched in the group 5 treatment. However, genes associated with the CAZy database, including carbohydrate esterases (CE), carbohydrate-binding modules (CBM), and polysaccharide lyases (PL) (Fig. 7b; Table S4E), and those associated with the VFDB and involved in defensive virulence factors (DVF) (Fig. 7d; Table S4F) were significantly enriched in the group 1 treatment. The genes associated with glycoside hydrolases (GH) (Fig. 7b) were significantly enriched in the group 9 treatment.

10.1128/msystems.00418-22.4 FIG S4 PCoA based on NR, CAZy, CARD, ARDB, VFDB, QS, PHI, and P450 database annotations among the group 1, group 5, and group 9 treatments. Download FIG S4, TIF file, 0.6 MB. Copyright © 2022 Guo et al. 2022 Guo et al. https://creativecommons.org/licenses/by/4.0/ This content is distributed under the terms of the Creative Commons Attribution 4.0 International license.

10.1128/msystems.00418-22.9 TABLE S4 (A) Number of reads from metagenomic shotgun sequencing of the three soil water treatment groups (group 1, group 5, and group 9). (B) Difference analysis of the metagenomic microbial function profiling in KEGG level 2 functional categories. (C) Difference analysis of the metagenomic microbial function profiling in KEGG level 3 functional categories. (D) Difference analysis of the metagenomic microbial function profiling based on comprehensive antibiotic resistance database (CARD). (E) Difference analysis of the metagenomic microbial function profiling based on carbohydrate active enzymes (CAZyme). (F) Difference analysis of the metagenomic microbial function profiling based on virulent factor annotation (VFDB). Download Table S4, XLSX file, 0.05 MB. Copyright © 2022 Guo et al. 2022 Guo et al. https://creativecommons.org/licenses/by/4.0/ This content is distributed under the terms of the Creative Commons Attribution 4.0 International license.

Based on the level 2 KEGG functional categories, pathways involved in infectious diseases, environmental adaptation, cell growth and death (apoptosis), translation, and so on were significantly enriched under both low- and high-soil-moisture conditions (Fig. 7e; Table S4B). Pathways involved in cell motility, aging, infectious diseases, transport, and catabolism were significantly enriched under high-soil-moisture conditions (Fig. 7e). Pathways involved in lipid metabolism, terpenoid and polyketide metabolism, energy metabolism, and so on were significantly enriched under low-moisture conditions (Fig. 7e). However, pathways involved in the biosynthesis of secondary metabolites and terpenoid and polyketide metabolism were significantly enriched under proper-moisture conditions (Fig. 7e). Specifically, a total of 415 pathways in KEGG level 3 were noted (Fig. S5; Table S4C), of which 207 pathways (accounting for 50%) showed significant differences among the three soil moisture states (Fig. S5; Table S4C). A total of 27 pathways (Fig. S5; Table S4C) were significantly enriched in the group 5 treatment. Among them, those involved in the biosynthesis of secondary metabolites (neomycin, kanamycin, and gentamicin; streptomycin; and staurosporine) and the metabolism of terpenoids and polyketides (biosynthesis of enediyne antibiotics and tetracycline) were significantly enriched in the group 5 treatment (Fig. 7f). A total of 30 pathways (Fig. S5; Table S4C), including plant-pathogen interactions and bacterial secretion systems (Fig. 7f), were significantly enriched in the group 1 and group 9 treatments. Independently, 50 pathways (Fig. S5; Table S4C), including apoptosis and bacterial invasion, were significantly enriched in the group 1 treatment. Seventeen pathways, including quorum sensing and bacterial chemotaxis (Fig. S5; Table S4C), were significantly enriched in the group 9 treatment. These data confirmed that the antibiotic biosynthesis-related genes were enriched under proper-moisture conditions, but pathogenicity-related genes were enriched under extreme-moisture conditions.

10.1128/msystems.00418-22.5 FIG S5 KEGG pathway enrichment and relative abundances of the genes involved in virulence among different levels of soil moisture. (a) Pathway enrichment plot displaying the metagenomic shotgun sequencing among the three treatments (group 1, group 5, and group 9). The ratio of the relative abundance of each pathway between groups is shown. Each circle in the plot represents one pathway. The circles of different colors represent the pathways enriched in the different treatments. The gray circles indicate that there was no significant difference among the three treatments (Nosig). The red circles indicate enrichment in group 1; orange, enrichment in group 1 and group 9 (Group 1_9); green, enrichment in group 5; blue, enrichment in group 9. The black circles indicate the other pathways for which there were significant differences among the three treatments but which did not belong to one of the above enrichment groups (others). All the pathways were evaluated using the Tukey HSD test. Download FIG S5, TIF file, 0.1 MB. Copyright © 2022 Guo et al. 2022 Guo et al. https://creativecommons.org/licenses/by/4.0/ This content is distributed under the terms of the Creative Commons Attribution 4.0 International license.

## DISCUSSION

Indigenous antagonistic soil microbiomes well adapted to their hosts and associated environments have been harnessed to control soilborne diseases (6, 9). Encouragingly, an increasing number of indigenous microbiomes have been identified with advances in DNA sequencing and microbial culturing techniques, such as simplified root-associated bacterial community identification based on host-mediated selection (29), keystone taxon identification by co-occurrence and network analysis (30, 31), and antagonistic microbiome identification based on network and potential function analysis (32). However, it is still a challenge to manage a reliable microbiome due to the complexity of the root-associated microbiome environment. Here, we identified the keystone species associated with root rot disease suppression and susceptibility using network analysis and revealed that soil moisture could influence these keystone species to affect microbial suppression.

### Soil moisture changes microbial suppression by modifying keystone species.

We found that the occurrence of root rot disease was aggravated by soil moisture stress conditions based on field and pot experiments (Fig. 1a and c). We further solidified the evidence that this kind of legacy effect from moisture on root rot disease was mediated by the soil microbiome (Fig. 1b). It was found that fungi modified by improper soil moisture (>95% or <75%), especially the soilborne pathogens *I. destructans* and *M. cucumerina* (24, 26, 28), played a key role in root rot disease occurrence (Fig. 2 and 3). Soil moisture-modified bacteria were divided into two opposite classes based on their correlation with root rot disease: the disease-suppressive microbiome (RN, RNP, MN, and RPN) and the disease-inductive microbiome (RP, IP, MP, RPP, and RNN) (Fig. 3). Combined with the results of the *in vitro* and *in planta* experiments, we confirmed that the synthetic disease-inductive microbiotas Syn-RP4 and Syn-MP8 could synergize with *I. destructans* and *M. cucumerina*, respectively, to aggravate root rot disease (Fig. 5). However, the synthetic disease-suppressive microbiota Syn-RN4, which is mainly derived from *Actinobacteria* and *Firmicutes* and frequently reported as an antagonist (4, 27, 28, 33), showed antagonistic activity against the two soilborne pathogens and disease-inductive microbiota Syn-RP4 (Fig. 5a) and then alleviated the root rot disease (Fig. 5f to h). Further *in planta* experiments confirmed that the unbalanced proportion of Syn-RN4 and Syn-RP4 according to their abundances in low- and high-moisture soil could aggravate root rot disease (Fig. 6).

Microbial disease resistance function changes caused by soil moisture were further supported by metagenomic analysis. The abundance of genes involved in the biosynthetic pathways of antibiotics was enriched under ideal soil moisture conditions (Fig. 7); however, genes involved in disease infection- or pathogenicity-related pathways were enriched under extreme soil moisture conditions (Fig. 7). These results were consistent with previous studies showing that disease-suppressive soils contain a wealth of antibiotic biosynthetic loci (4) and that antibiotic biosynthetic pathways are significantly enriched in soils associated with low potato common scab severity, but pathogen-related genes are upregulated in inductive soils (34). Overall, proper soil moisture management could maintain microbial suppression, but low or high soil moisture stress could enrich the disease-inductive microbiome to change soil from a disease-suppressive to a disease-inductive state.

### Soil moisture can directly modify soil microbial community and affect soil microbial suppression.

The responses of microbes in soil to moisture are changeable (35). Based on hierarchical clustering analysis (Fig. S2e and f), we found that low soil moisture had a stronger effect on the bacterial community than on the fungal community, but high soil moisture had a strong effect on the fungal community. These results were consistent with those of previous studies, which showed that stronger changes occurred in bacterial communities than in fungal communities under drought conditions (36) but that fungal community change was directly proportional to the precipitation gradient (37) and soil humidity (38). The difference in the sensitivity of fungi and bacteria to soil moisture resulted in a change in the soil disease suppression ability. At the community level, both low and high soil moisture markedly increased the richness and diversity in the fungal community but decreased those in the bacterial community and eventually caused a shift in soil microbes from a bacterium-dominated community to a fungus-dominated community, which implied a decrease in microbial suppression of root rot disease (39, 40). For bacteria, most of the top 10 bacterial phyla were significantly modified by soil moisture changes from low to high (Fig. S2f; Table S2C). Among them, desiccation-resistant bacteria (e.g., *Actinobacteria*, *Bacteroidetes*, *Cyanobacteria*, *Gemmatimonadetes*, and *Chloroflexi*) (12, 17, 18, 41–43) were enriched at low soil moisture (<65%) (Fig. S2f; Table S2C), and desiccation-sensitive bacteria (e.g., *Proteobacteria*) (19) were favored at high soil moisture (<65%) (Fig. S2f; Table S2C). However, antagonistic bacteria from *Actinobacteria* and *Firmicutes* were enriched at the proper soil moisture (75 to 85%) (Fig. S2f; Table S2C). For fungi, Ascomycota and Basidiomycota, which can survive under dry conditions and grow vigorously under high humidity (44), were significantly enriched under low and high soil moisture (Fig. S2e; Table S2D). Previous reports demonstrated that most soilborne fungal pathogens isolated from *P. notoginseng* were derived from Ascomycota, including *Ilyonectria*, Fusarium, and *Rhizoctonia* (24, 26, 28, 45). In this study, the positive correlation between the abundance of Ascomycota and root rot disease incidence confirmed the role of Ascomycota in disease occurrence (Fig. S2e; Table S2D). In particular, low soil moisture enriched the abundance of *Monographella*, but high soil moisture favored the growth of *Ilyonectria*, confirming the point that soil moisture governs the rate of reproduction of fungal pathogens.

### Soil moisture stress suppressed antagonistic microbes to decrease soil microbial suppression.

The depletion of the disease-suppressive microbiome and antibiotic biosynthesis genes under extreme moisture conditions (>95% or <75%) demonstrated that the soil state was unsuitable for the growth of beneficial pathogen-antagonistic bacteria. Metagenomic data indicated that pathways involved in environmental adaptation, cell growth and death (apoptosis), and oxidative stress (ascorbate and aldarate metabolism, oxidative phosphorylation) were significantly enriched under both low- and high-soil-moisture conditions (Fig. 7e), which implied that the soil reached a stress state. Emerging evidence suggests that changes in soil physicochemical properties, such as N, pH, and electrical conductivity (EC), could enrich soil microbial genes involved in the stress response and suppress beneficial microbes but enrich pathogens (28, 46). Many beneficial soil microorganisms are generally saprophytic, and their growth relies on the soil microenvironment (47). When the soil state was stressed by soil moisture, the abundance of antagonistic genera in the disease-suppressive microbiome, including *Bacillus*, *Paenibacillus*, *Arthrobacter*, and Pseudomonas, was significantly suppressed (Fig. 3), subsequently affecting the antagonistic activity of the microbiome. Numerous studies have revealed that antagonistic activities occur between bacteria and fungi or oomycetes in soil (48, 49). *In vitro* and *in planta* experiments on the antagonistic activity of the disease-suppressive microbiota Syn-RN4 against the disease-inductive microbiota Syn-RP4 as well as the soilborne pathogens (Fig. 5 and 6) further confirmed that low and high soil moisture suppressed the disease-suppressive microbiome and then decreased the soil antagonistic activity against soilborne pathogens.

### Soil moisture stress could enrich soilborne pathogens through host-specific selection to decrease soil microbial suppression.

Many studies report that when soil nutrients or the microenvironment is unsuitable, host-specific pathogens tend to be enriched due to host selection (28, 47). Soil biotic and abiotic stresses have been reported to change the metabolites of plants and thereby affect the secretion of root exudates, which modifies the rhizosphere microbial community (11, 42, 50, 51). Previous studies demonstrated that soil moisture change had a stronger impact on the root endosphere and rhizosphere than on the surrounding soil (11, 42), implying that the effects of soil moisture on the root microbiome could be mediated by plants. In this study, the abundances of the disease-inductive microbiome and genes involved in pathogenicity were significantly enriched in rhizosphere soil under low- and high-soil-moisture conditions (Fig. 2; Fig. 7f). These results showed that *P. notoginseng* could specifically select keystone pathogenic taxa under stressed soil moisture conditions. In particular, the soilborne pathogens *Monographella* and *Ilyonectria*, which could be specifically stimulated by root exudates of *P. notoginseng* (52), were selectively enriched under low- and high-soil-moisture conditions, respectively (Fig. 2). Moreover, *P. notoginseng* grows poorly under low- and high-soil-moisture conditions and is inductive to the typical soilborne opportunistic fungi *Monographella* and *Ilyonectria* (Fig. 1c), which may in turn facilitate the buildup of host-specific pathogens in soil. Therefore, soil moisture-mediated root metabolite shifts and their relationship with the rhizosphere microbial composition deserve further research.

### Conclusions.

Soil moisture is a key factor in balancing disease-suppressive and disease-inductive microbiomes. Proper soil moisture management could maintain microbial disease suppression, but low- and high-soil-moisture stresses could have negative effects on suppression and aid accumulation of host-specific soilborne pathogens to aggravate root rot disease. This finding could provide new strategies for sustainable control of root rot disease by enriching the indigenous disease-suppressive microbiome through soil moisture management.

## MATERIALS AND METHODS

### Experimental design and growth conditions.

Pot and field experiments were conducted in a plastic greenhouse. *P. notoginseng* is a shade-demanding plant. Therefore, the plastic house was shaded with a polyethylene net that allowed ~10% light transmission, and the temperature was kept below 28°C. Healthy seedlings were planted in soil at 35.3% (wt/wt) field capacity (FC) (Table S5).

10.1128/msystems.00418-22.10 TABLE S5 Chemical characteristics of the soil used in pot and field experiments. Download Table S5, XLSX file, 0.01 MB. Copyright © 2022 Guo et al. 2022 Guo et al. https://creativecommons.org/licenses/by/4.0/ This content is distributed under the terms of the Creative Commons Attribution 4.0 International license.

For the field experiment, three levels of soil moisture, including 47.6 to 60%, 57.5 to 75%, and 61.6 to 95% FC, were used, and their effect on root rot disease was evaluated (Fig. S1a). Soil moisture was manually adjusted to an appropriate level every 48 h and monitored with TDR 100 soil moisture meter (Spectrum Technologies, Inc., USA) (53). The experiment was set up with four replications for each treatment using a randomized complete block design. The area of each plot was 1.5 m in length by 1.2 m in width, and plots were separated with a 1.0 m buffer area. The plots and buffer areas were separated with plastic film to avoid interference. A total of 120 seedlings were planted at a density of 15 cm by 10 cm in each plot. Seedling mortality was observed nine times under the soil moisture treatments, which were continuously applied for 7 months. All seedlings in each plot were harvested to assess the incidence of root rot disease after 7 months (Fig. S1a). Then, the soil in each plot was evenly divided into two subsamples for seedling reestablishment analysis as described in a previous study (28). One subsample was sterilized with steam at 90°C for 15 min, and the other was not sterilized. The seedlings were replanted at the above-mentioned density, and the seedling emergence rate was investigated to estimate the effect on the reestablishment of *P. notoginseng*. The same moisture treatment was used in all plots throughout the course of the seedling reestablishment experiment. Seedling emergence rate in the untreated soil was measured when the emergence rate exceeded 70% in the sterilized soil.

Due to the difficulty in accurately controlling soil moisture in the field plot, pot experiments with nine levels of soil moisture (55 to 60%, 60 to 65%, 65 to 70%, 70 to 75%, 75 to 80%, 80 to 85%, 85 to 90%, 90 to 95%, and 95 to 100% of FC), ranging from drought to high soil moisture, were designed to evaluate the effect of soil moisture changes on the occurrence of soilborne disease in the plastic house (Fig. S1b). The soil used in the pot experiment was consistent with that used in field experiments. Each pot (40 cm by 30 cm by 15 cm) was filled with 14 kg of soil. Nine seedlings were transplanted into each pot at a density of 15 cm by 10 cm. All pots were placed in the plastic house (with a 12-h photoperiod with temperatures maintained below 28°C) and rotated every week. Soil moisture was maintained by manual irrigation every 48 h and monitored using the accurate weight method (54). After 90 days of soil moisture control, all the plants in each container were harvested to assess the incidence of soilborne disease, and the rhizosphere soil and bulk soil were collected for further experiments. The incidence of soilborne disease was investigated as described in a previous study (24). The pathogenic fungi were isolated from roots showing typical symptoms of root rot disease and identified according to the morphology of the mycelia and spores, combined with Koch’s postulates (26). The rhizosphere and bulk soil were collected according to a previously described method (28). Briefly, the roots were carefully removed from the soil, the large particles of soil loosely attached to the plant roots were removed by gentle shaking, and the soil attached to the roots was collected and evenly divided into two subsamples. One subsample was stored at −80°C until DNA extraction and was used for microbial community and metagenomic shotgun sequencing analysis. The other subsample was stored at 4°C and used for the isolation of microbes. The bulk soil was also collected, sieved through a 40-mesh screen to remove fibrous root debris, and then stored at room temperature for use as continuous cropping soils in further experiments.

### Soil physicochemical analyses.

Soil pH and EC were determined using a mixture of soil and deionized water free of CO_2_ at a ratio of 1:2.5 (wt/vol) with an Inolab pH/Cond 720 instrument (WTW, Koblenz, Germany) (28). Soil total carbon (TC), total nitrogen (TN), organic matter (OM), available phosphorus (AP), available potassium (AK) and available nitrogen (AN) were assayed according to the methods described in previous studies (28). Three technical replicates for each soil sample were performed.

### Evaluation of the effects of soil moisture on the microbial community.

All 27 rhizosphere soil samples with nine levels of soil moisture were selected for microbial community analysis through 16S rRNA gene and internal transcribed spacer (ITS) amplicon sequencing. Genomic DNA was extracted using the PowerSoil DNA isolation kit (MO BIO Laboratories, Inc., USA) according to the instruction manual. The V4-V5 region of the bacterial 16S rRNA was amplified with universal primers (F515, 5′-GTGCCAGCMGCCGCGG-3′, and R907, 5′-CCGTCAATTCMTTTRAGTTT-3′) (55). The fungal ITS2 genes were amplified with universal primers (ITS3-2024F, 5′-GCATCGATGAAGAACGCAGC-3′, and ITS4-2409R, 5′-TCCTCCGCTTATTGATATGC-3′) (56). The amplified PCR products were further purified and qualified by Qubit and qPCR. A total of 5.1 Gb of raw reads was obtained after sequencing with a HiSeq PE2500 sequencer (Illumina, USA). The barcode and primer sequences were removed from the raw reads based on their unique barcodes and truncated. The sequences were assembled according to a default script in QIIME (57). Through quality filtering and chimera removal (58), effective sequences were generated and then used to identify operational taxonomic units (OTUs) based on 97% pairwise identity by the UPARSE-OTU algorithm in USEARCH software (59) and perform species annotation using the UCLUST algorithm (60). The taxonomic assignment of each OTU was determined by using the Unite database and Silva database for bacteria and fungi, respectively (61). Alpha diversity indices (Chao1, coverage, and the Shannon and Simpson indices) were calculated with Mothur v. 1.34.4 (62). The relative abundances of individual OTUs were calculated, and the results were used for further statistical analysis. A total of 2,102,299 reads from 27 samples of 16S rRNA were obtained; the number of reads per sample ranged from 52,801 to 105,156, with an average of 77,863 (Table S2A). The number of OTUs with a sequence identity of 97% ranged from 4,123 to 7,778 (Table S2A). After sequencing of the ITS region, a total of 2,817,543 reads were obtained; the number of reads per sample ranged from 83,219 to 141,228, with an average of 104,353 per sample (Table S2B). All the fungal tags were clustered into between 359 and 972 OTUs at 97% sequence identity (Table S2B).

### Network analysis of the microbial community associated with soilborne disease.

To explore the effect of bacterial and fungal community changes on soilborne disease, the correlations of alpha and beta diversity and the relative abundances of bacteria and fungi at the phylum and genus levels with soilborne disease incidence were analyzed. Then, the core bacterial microbiome associated with soilborne disease was inferred through a correlation analysis between the relative abundance of soil moisture-modified bacteria and the incidence of soilborne disease as well as the relative abundances of two fungal pathogens, *Ilyonectria* and *Monographella*. First, all the bacteria significantly modified by soil moisture (*P < *0.05) were selected for further correlation analysis. Second, a Pearson correlation analysis between the soilborne disease incidence and the relative abundance of all soil moisture-modified bacteria as well as *Ilyonectria* and *Monographella* was carried out. All the genera that showed a significantly positive correlation with soilborne disease (*P < *0.05) were defined as disease-inductive microbiota (RP). All the genera that showed a significantly negative correlation with soilborne disease (*P < *0.05) were defined as disease-suppressive microbiota (RN). Third, a correlation analysis was performed between other bacterial genera, which were significantly modified by soil moisture but not directly correlated with soilborne disease, and the genera in the RN or RP microbiota as well as *Ilyonectria* and *Monographella*.

### Isolation, identification, and functional analysis of the soil bacteria.

The rhizosphere bacteria were isolated using a series of media, including tryptic soy agar (TSA), 1/10 TSA (the TSA medium was diluted 10 times), R2A agar, 1/10 R2A (the R2A medium was diluted 10 times), LB nutrient agar, and Yeast Extract Malt Extract Agar (ISP medium 2), with gradient dilution according to previously described methods (29, 63). All the isolates were purified on LB medium and then stored at −20°C. The bacterial 16S rRNA genes of all the isolates were amplified with universal primers (27F, AGA GTT TGA TCM TGG CTC AG, and 1492R, GGT TAC CTT TGT TAC GAC TT) (64) and then identified by alignment against published 16S rRNA gene sequences from the NCBI (https://blast.ncbi.nlm.nih.gov/Blast.cgi). A phylogenetic tree was constructed with MEGA software (MEGA 6.06) based on the neighbor-joining method.

The antagonistic activity of all isolates against soilborne pathogens (*I. destructans* and *M. cucumerina*) was tested on potato dextrose agar (PDA) according to a previously described method (26). Briefly, a mycelial block of the pathogen with a 5-mm diameter was placed in the middle of a petri dish (9-cm diameter) and vertically surrounded by four test isolates at a distance of 25 mm. Petri dishes inoculated with pathogenic mycelium without test isolates were used as controls. All petri dishes were incubated in the dark at 25°C. All the bacterial isolates were tested three times with three replicates. Antagonistic activity was calculated according to the methods described in a previous report (26). The antagonistic activity of the tested isolates against the two pathogens ranged from low to high and was divided into four levels: <0 (−), 0 to 25% (+), 25 to 50% (++) and >50% (+++).

To further verify the functions of the keystone species in soilborne disease occurrence, a total of 16 isolates, including four isolates from the RN microbiota (*Arthrobacter*, *Bacillus*, *Paenibacillus*, and *Sporosarcina*), four isolates from the RP microbiota (*Dyadobacter*, *Sphingobacterium*, *Sphingobium*, and *Sphingopyxis*), and eight isolates from the MP microbiota (*Rhizobium*, *Bradyrhizobium*, *Aminobacter*, *Phyllobacterium*, *Olivibacter*, *Pseudoxanthomonas*, Mycobacterium, and *Ramlibacter*), were selected to form three synthetic microbiota (Syn-RN4, Syn-RP4, and Syn-MP8) for further study (Table S3B).

The antagonistic activity of Syn-RN4 against Syn-RP4 and soilborne pathogens (*I. destructans* and *M. cucumerina*) was tested on PDA. Briefly, a mycelial block of the pathogen with a 5-mm diameter was placed along the edge of a petri dish (9-cm diameter), and two lines (four points on each line) of Syn-RN4 or Syn-RP4 were vertically aligned at distances of 45 mm and 60 mm, respectively, from the mycelial block. Petri dishes inoculated with pathogenic mycelium but without a microbiota were used as controls. All petri dishes were incubated in the dark at 25°C. All the bacterial isolates were tested three times with three replicates. The colony diameters of *I. destructans* and *M. cucumerina* as well as Syn-RN4 and Syn-RP4 were measured. The antagonistic activity was also calculated using the methods described above.

The effects of the three synthetic microbiotas on soilborne disease were tested in pot experiments. The bulk soil from nine soil moisture treatments in the pot experiment was mixed to form a continuous cropping soil and then separated into four subsamples. The first subsample was nontreated continuous cropping soil (CS); the second subsample was heat treated at 80°C for 2 h (S) according to the methods of a previous study (6); the third subsample was heat treated at 80°C for 2 h and then inoculated with a spore suspension of *I. destructans* at a concentration of 3 × 10^5^ spores/g soil (SI); and the fourth subsample was heat treated at 80°C for 2 h and then inoculated with a spore suspension of *M. cucumerina* at a concentration of 2.5 × 10^5^ spores/g soil (SM). The soil was separated into plastic pots (9 cm by 33 cm^2^), with each pot containing 100 g of soil. Bacterial suspensions of all the selected isolates were prepared according to previously described methods (9). Briefly, bacterial suspensions of individual isolates or mixed microbiotas inoculated into pots were adjusted to 1 ~ 3 × 10^7^ CFU/g soil. A pot inoculated with sterile water served as the control treatment. We further added the Syn-RP4 individually or mixed with Syn-RN4 to CS soil according to their relative abundances under low soil moisture (group 1, 55 to 60% of FC), proper soil moisture (group 5, 75 to 80% of FC), and high soil moisture (group 9, 95 to 100% of FC) (based on 16S rRNA amplicon sequencing). There were four replicates of each treatment. Two days after the inoculation of the above-mentioned bacterial suspension, 10 surface-sterilized seeds were planted in each pot, and then all the pots were incubated in a plastic house under the same conditions. The seedling emergence rate was recorded periodically after 40 days.

### Metagenomic analysis.

Based on the above-mentioned results of the microbial community analysis of the nine treatment groups, three soil moisture treatments, including the drought treatment (group 1, 55 to 60% of FC), suitable-soil-moisture treatment (group 5, 75 to 80% of FC), and high-soil-moisture treatment (group 9, 95 to 100% of FC), were selected for further metagenomic shotgun sequencing to evaluate the functional changes in the microbial community. Total genomic DNA was extracted from nine samples using the E.Z.N.A. soil DNA kit (Omega Bio-Tek, Norcross, GA, USA) according to the manufacturer’s instructions. The concentration and purity of the extracted DNA were determined with TBS-380 and NanoDrop 2000 instruments, respectively. The DNA extract quality was checked on a 1% agarose gel. The DNA extract was fragmented to an average size of approximately 300 bp using Covaris M220 (Gene Company Limited, China) for paired-end library construction. A paired-end library was constructed using NEXTflex Rapid DNA-Seq kits (Bioo Scientific, Austin, TX, USA). Adapters containing the full complement of sequencing primer hybridization sites were ligated to the blunt ends of the fragments.

Paired-end sequencing was performed on an Illumina NovaSeq 6000 (Illumina Inc., San Diego, CA, USA) platform at Majorbio Bio-Pharm Technology Co., Ltd. (Shanghai, China) using NovaSeq reagent kits according to the manufacturer’s instructions (www.illumina.com). Approximately 86.8 Gb of raw reads was obtained from the 9 samples. Adapter sequences were filtered from the raw sequences using SeqPrep software (https://github.com/jstjohn/SeqPrep), and reads with a length of <50 bp, with a quality value <20, or with N bases were removed by Sickle (https://github.com/najoshi/sickle). A total of 1,222,905,692 paired-end sequences were obtained, with an average of 135,878,410 clean reads for each soil sample (minimum = 127,908,468; maximum = 145,117,114). A total of 1,202,657,663 optimized sequence reads were obtained by BWA software (https://github.com/lh3/bwa) by removing the host sequences after alignment with *P. notoginseng* DNA sequences. These reads were assembled by MEGAHIT (https://github.com/voutcn/megahit) based on a succinct de Bruijn graph (65), and a total of 15,450,314 contigs were obtained. The resulting contigs >300 bp in length were selected to predict open reading frames (ORFs) using MetaGene (66), and a total of 20,063,460 ORFs were obtained (Table S4A).

All genes of >100 bp were selected for translation into putative amino acid sequences. All the predicted genes were clustered with 95% sequence identity (90% coverage) using CD-HIT software (http://www.bioinformatics.org/cd-hit/) (67). The longest sequences from each cluster were selected as the representative sequences, from which a nonredundant gene catalog was constructed. The high-quality reads obtained after each sample read underwent quality control were mapped to the representative sequences with 95% identity using SOAPaligner (https://github.com/ShujiaHuang/SOAPaligner) (68). The gene abundance was calculated using the reads per kilobase per million mapped reads (RPKM) method (69).

The representative sequences of the nonredundant gene catalog were aligned with the NR, KEGG, CAZy, CARD, VFDB, PHI, QS, and P450 databases to perform species and gene functional annotation. Briefly, a nonredundant gene catalog was aligned against the NCBI NR database, with an E value cutoff of 1e−5, using BLASTp (version 2.2.28+; http://blast.ncbi.nlm.nih.gov/Blast.cgi) (70). The KEGG annotation was conducted using BLASTp against the Kyoto Encyclopedia of Genes and Genomes database (https://www.genome.jp/kegg/), with an E value cutoff of 1e−5 (71). Comprehensive antibiotic resistance annotation was performed using BLASTp against the CARD database (E value ≤ 1e−5). Carbohydrate-active enzyme annotation was conducted using the hmmscan tool against the CAZy databases (E value ≤ 1e−5). Antibiotic resistance annotation was conducted using a BLASTP search (version 2.2.28+) against the ARDB database (http://ardb.cbcb.umd.edu/), with an E value cutoff of 1e−5. The virulent factor annotation was conducted using a BLASTp search against the VFDB database (http://www.mgc.ac.cn/VFs/), with an E value cutoff of 1e−5, and the quorum sensing and cytochrome P450 annotation was performed based on Diamond software using a BLASTp search against the QS and P450 databases, respectively (E value ≤ 1e−5). The putative amino acid sequences, which were translated from the gene catalog, were aligned against the PHI database using BLASTp (E value ≤ 1e−5). The relative abundances of all the species, KEGG orthology (KO) functional categories, KEGG pathways, enzymes, and genes were calculated for further analysis.

### Statistical analysis.

SPSS version 19.0 software (Chicago, USA) was used for the general statistical analyses. The normality of the data distribution and homogeneity of variance were checked before the statistical analyses. Significance among the treatments was tested by one-way analysis of variance (ANOVA) with Tukey’s honestly significant difference (HSD) test (*P < *0.05). A Pearson correlation analysis was carried out between the incidence of root rot disease and the relative abundance of microbes. The Bray-Curtis metric was calculated using the vegdist function from the R package vegan (v. 2.4-4). Principal-coordinate analysis (PCoA) was performed using the prcomp function from the R package “stats” with the Bray-Curtis metric and weighted UniFrac distances. A heat map was calculated within R software using the pheatmap package. The correlation network was visualized using Cytoscape v. 3.6.1.

### Data availability.

The Illumina sequencing reads of the 16S rRNA gene and ITS gene for 27 rhizosphere soil samples have been deposited in the NCBI BioProject databases with accession codes PRJNA601893. Data supporting the findings of this work are presented in the paper and the supplemental material. All data are available from the corresponding author upon request.
