# Supplementary figures and images for: Moisture Controls the Suppression of Panax notoginseng Root Rot Disease by Indigenous Bacterial Communities
Source: mSystems. 2022 Aug 24;7(5):e00418-22. doi: 10.1128/msystems.00418-22 (PMC9600642; doi:10.1128/msystems.00418-22)

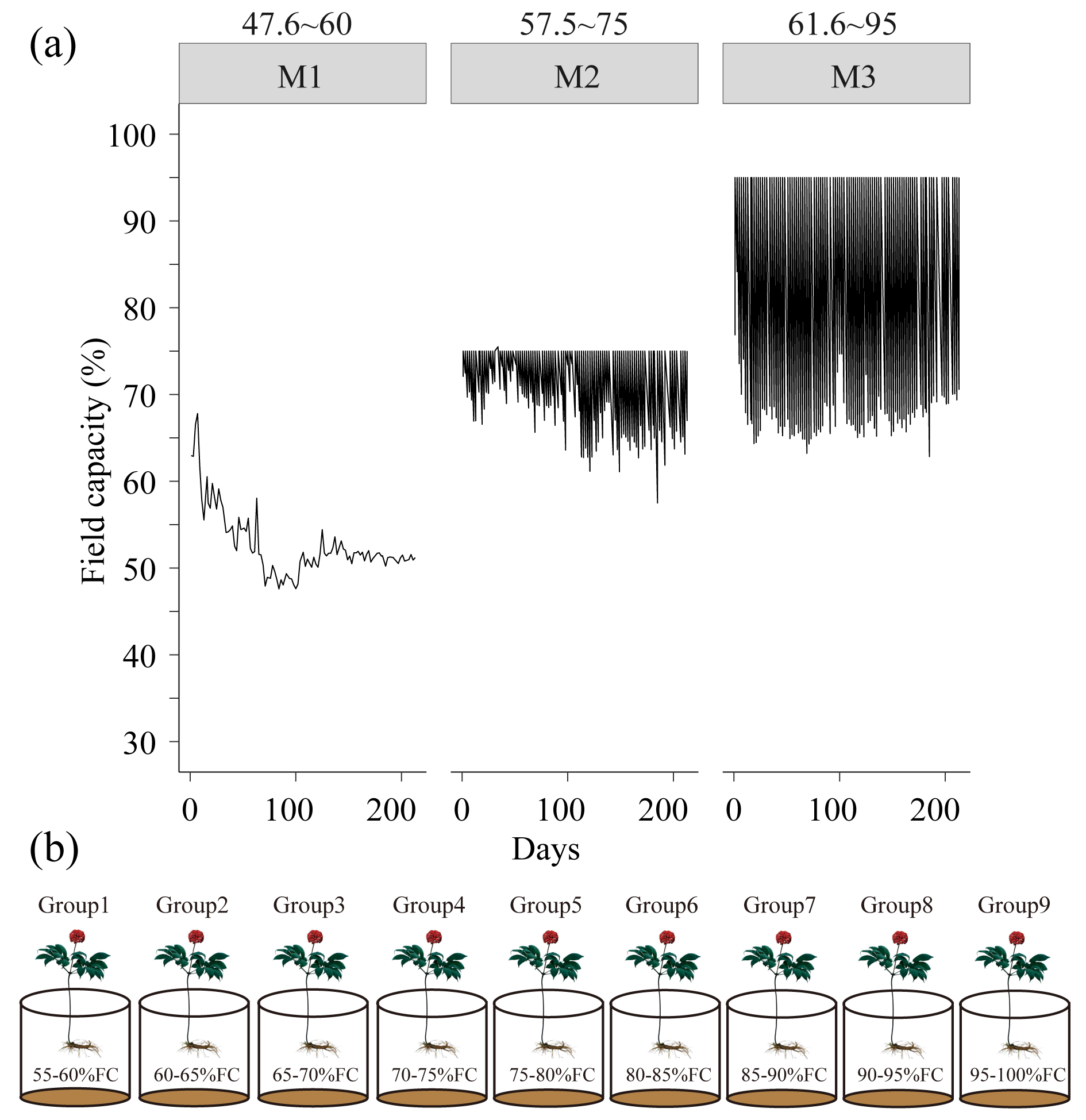

Supplement: FIG S1 [file msystems.00418-22-s0001.tif]

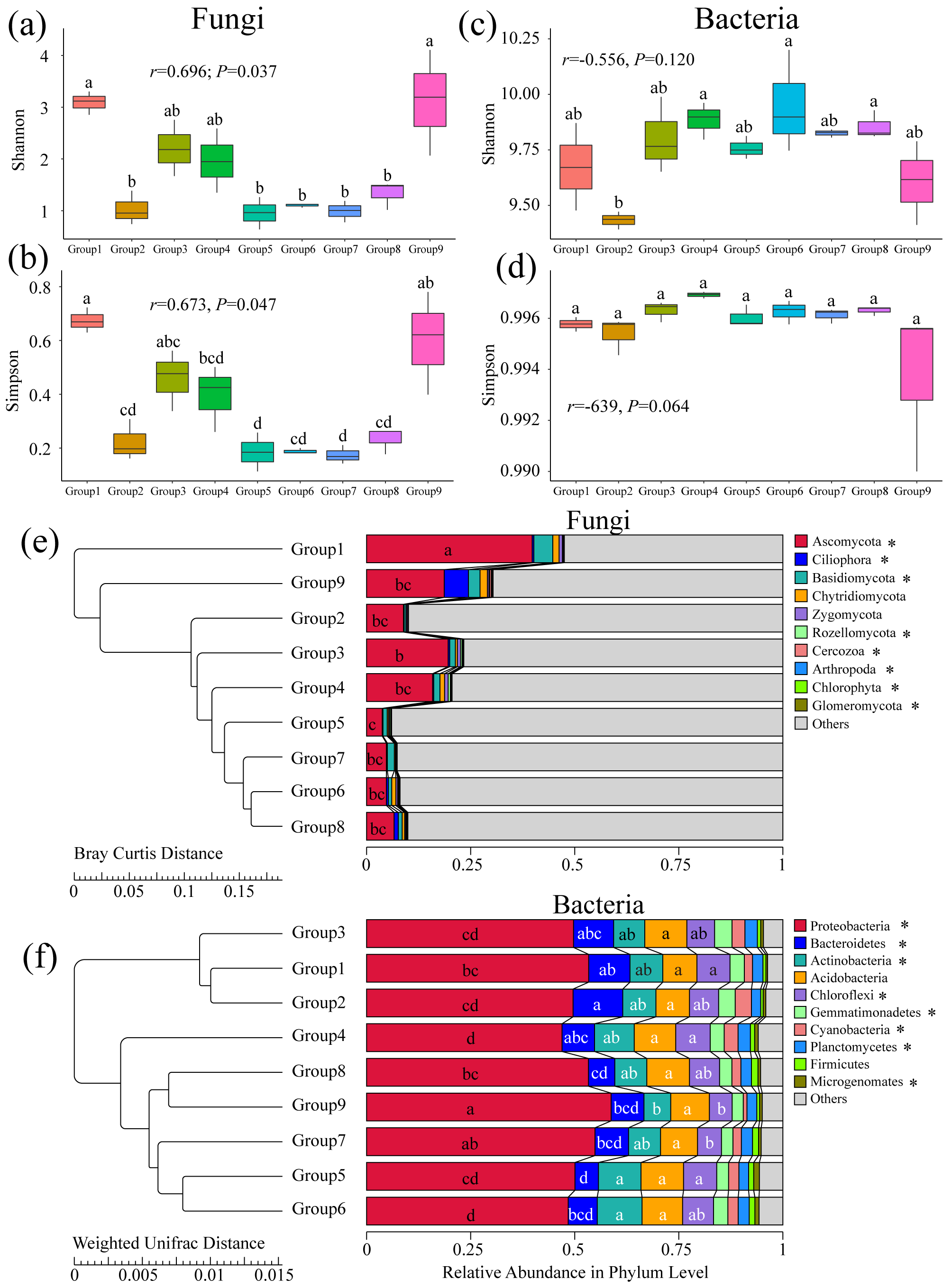

Supplement: FIG S2 [file msystems.00418-22-s0002.tif]

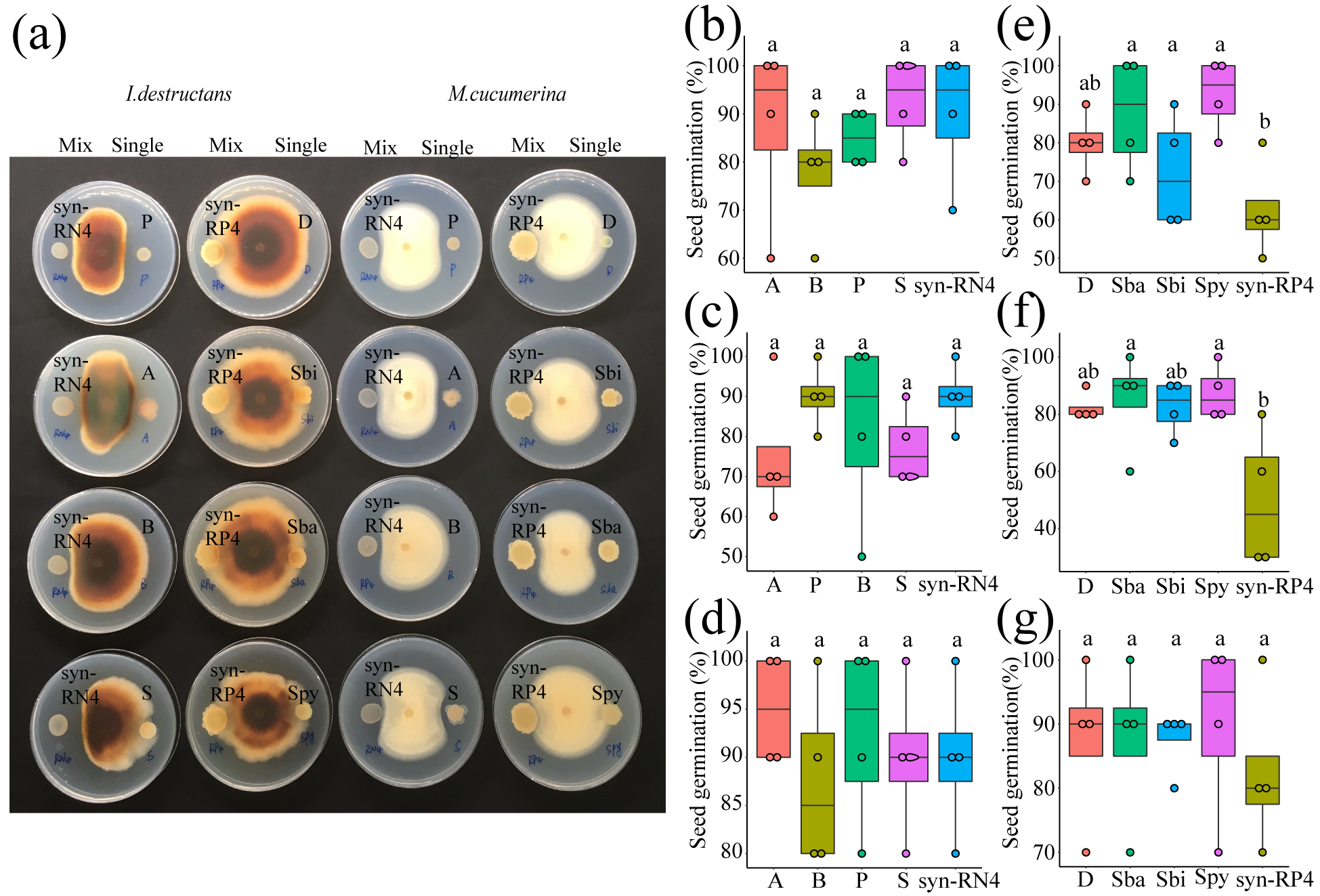

Supplement: FIG S3 [file msystems.00418-22-s0003.tif]

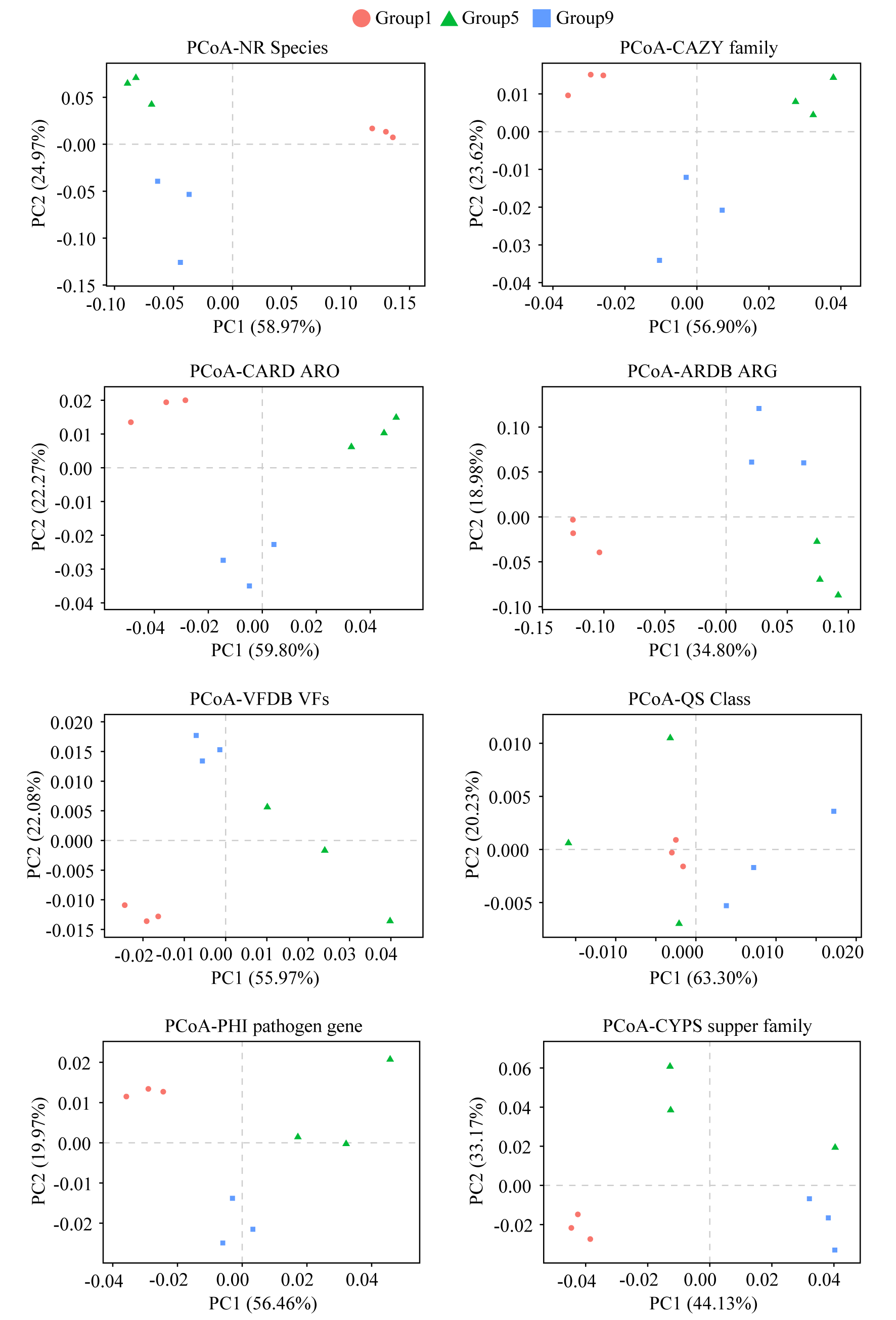

Supplement: FIG S4 [file msystems.00418-22-s0004.tif]

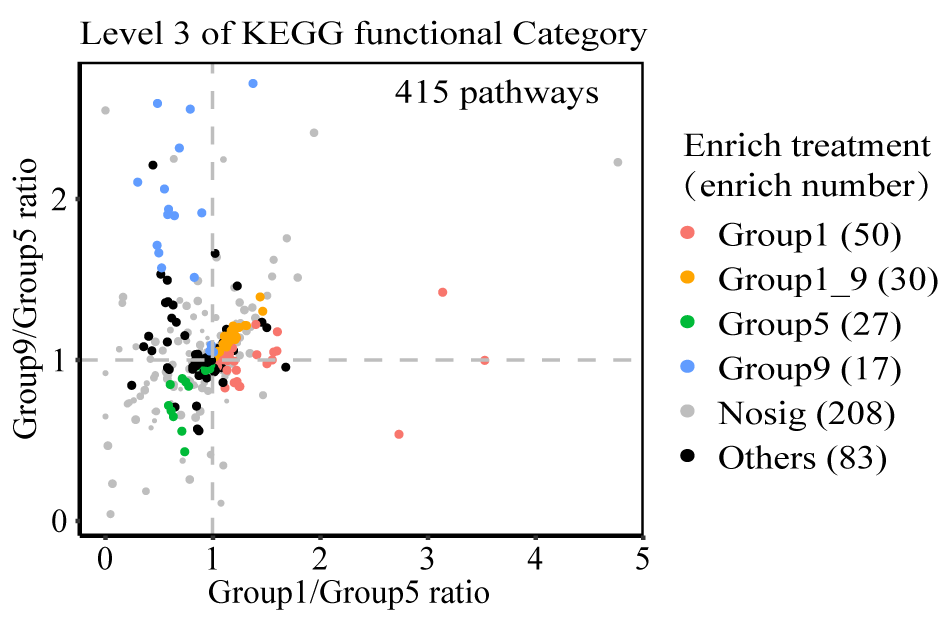

Supplement: FIG S5 [file msystems.00418-22-s0005.tif]
